# The miR-182-5p/FGF21/acetylcholine axis mediates the crosstalk between adipocytes and macrophages to promote beige fat thermogenesis

**DOI:** 10.1172/jci.insight.150249

**Published:** 2021-09-08

**Authors:** Wen Meng, Ting Xiao, Xiuci Liang, Jie Wen, Xinyi Peng, Jing Wang, Yi Zou, Jiahao Liu, Christie Bialowas, Hairong Luo, Yacheng Zhang, Bilian Liu, Jingjing Zhang, Fang Hu, Meilian Liu, Lily Q. Dong, Zhiguang Zhou, Feng Liu, Juli Bai

**Affiliations:** 1 National Clinical Research Center for Metabolic Diseases, Metabolic Syndrome Research Center, Key Laboratory of Diabetes Immunology, Ministry of Education, and Department of Metabolism and Endocrinology, The Second Xiangya Hospital of Central South University, Changsha, China.; 2 Department of Pharmacology and; 3 Greehey Children’s Cancer Research Institute, University of Texas Health at San Antonio, San Antonio, Texas, USA.; 4 Division of Plastic Surgery, Department of Surgery, Albany Medical Center, Albany, New York, USA.; 5 Department of Biochemistry and Molecular Biology, School of Basic Medical Sciences, Xinjiang Medical University, Ürümqi, China.; 6 Department of Biochemistry and Molecular Biology, University of New Mexico School of Medicine, Albuquerque, New Mexico, USA.; 7 Department of Cell Systems and Anatomy, University of Texas Health at San Antonio, San Antonio, Texas, USA.

**Keywords:** Endocrinology, Metabolism, Adipose tissue, Cellular immune response, Obesity

## Abstract

A dynamically regulated microenvironment, which is mediated by crosstalk between adipocytes and neighboring cells, is critical for adipose tissue homeostasis and function. However, information on key molecules and/or signaling pathways regulating the crosstalk remains limited. In this study, we identify adipocyte miRNA-182-5p (miR-182-5p) as a crucial antiobesity molecule that stimulated beige fat thermogenesis by promoting the crosstalk between adipocytes and macrophages. miR-182-5p was highly enriched in thermogenic adipocytes, and its expression was markedly stimulated by cold exposure in mice. In contrast, miR-182-5p expression was significantly reduced in adipose tissues of obese humans and mice. Knockout of miR-185-5p decreased cold-induced beige fat thermogenesis whereas overexpression of miR-185-5p increased beiging and thermogenesis in mice. Mechanistically, miR-182-5p promoted FGF21 expression and secretion in adipocytes by suppressing nuclear receptor subfamily 1 group D member 1 (Nr1d1) at 5′-UTR, which in turn stimulates acetylcholine synthesis and release in macrophages. Increased acetylcholine expression activated the nicotine acetylcholine receptor in adipocytes, which stimulated PKA signaling and consequent thermogenic gene expression. Our study reveals a key role of the miR-182-5p/FGF21/acetylcholine/acetylcholine receptor axis that mediates the crosstalk between adipocytes and macrophages to promote beige fat thermogenesis. Activation of the miR-182-5p–induced signaling pathway in adipose tissue may be an effective approach to ameliorate obesity and associated metabolic diseases.

## Graphical abstract

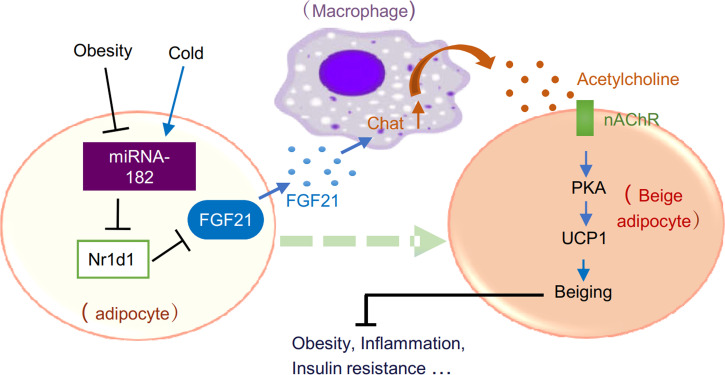

## Introduction

Excess expansion of white adipose tissue (WAT) due to positive energy balance and/or defects in thermogenesis in beige fat and brown adipose tissue (BAT) are associated with obesity and various metabolic diseases, such as insulin resistance and type 2 diabetes (1, 2). The adipose tissue microenvironment (ATME), which is modulated by signaling pathway crosstalk between adipocytes and neighboring cells, such as resident or infiltrated macrophages, has a great impact on adipose tissue function and systemic metabolic homeostasis (3, 4). However, the precise mechanisms by which adipocytes crosstalk to macrophages in adipose tissue remain to be further elucidated.

FGF21, which is mainly produced in the liver and to a lesser extent in adipose tissue, muscle, and pancreas, has various metabolically beneficial effects, such as reducing adiposity and hepatic triglyceride content, promoting thermogenesis and energy expenditure, and improving insulin sensitivity (5). Various studies have demonstrated that the physiological roles of FGF21 are mediated by its endocrine, autocrine, and/or paracrine actions. FGF21-deficient mice exhibit decreased cold-induced thermogenic gene expression in WAT but not BAT (6). It has also been found that adipose tissue– but not liver-derived FGF21 may regulate beige fat thermogenesis through a paracrine action (6, 7). However, how adipose FGF21 is regulated to exert its paracrine action and induce beige cell thermogenesis remains elusive.

MicroRNAs (miRNAs) are endogenous, noncoding small RNAs that posttranscriptionally regulate gene expression and have been shown to have important roles in numerous disease processes (8). In adipose tissues, miRNAs play an important role in regulating the pathways that control a range of processes, including adipocyte differentiation, insulin signaling, and glucose homeostasis (8). Recent studies have demonstrated that some miRNAs directly target and regulate thermogenic genes of brown adipocyte development and the beiging of white adipocytes (9–11). Among adipose tissue–expressed miRNA, miR-182-5p is highly enriched in differentiated brown adipocytes compared with nondifferentiated cells (12). However, whether and how miR-182-5p is involved in regulating the beiging progress of WAT is largely unknown.

In the current study, we show that adipose miR-182-5p levels were negatively associated with obesity in mice and humans. Adipose miR-182-5p expression was induced by cold exposure in mice, and its deficiency suppressed cold-induced expression of genes involved in mitochondrial biogenesis and thermogenesis. On the other hand, overexpression of miR-182-5p in adipose tissue promoted beiging of WAT and thermogenesis, improved energy homeostasis, and resisted diet-induced obesity. By inhibiting nuclear receptor subfamily 1 group D member 1 (Nr1d1), adipose miR-182-5p increased FGF21 expression and secretion, which in turn stimulated the production and secretion of acetylcholine in macrophages. Acetylcholine potentiated nicotinic acetylcholine receptor–mediated (nAChR-mediated) activation of the PKA signaling pathway in adipocytes and thus increased thermogenic gene expression. Our study identifies a potentially novel miR-182-5p/FGF21/acetylcholine/PKA signaling circuit that mediates the crosstalk between adipocytes and adipose tissue–resident macrophages to promote beige fat development and counteract overnutrition-induced obesity. Thus, upregulation of the miR-182-5p/FGF21/acetylcholine/PKA axis may be an effective approach to ameliorate obesity-induced metabolic diseases.

## Results

### miR-182-5p expression in WAT is negatively correlated with obesity in mice and humans but is stimulated by cold exposure in mice.

**Figure 1:**
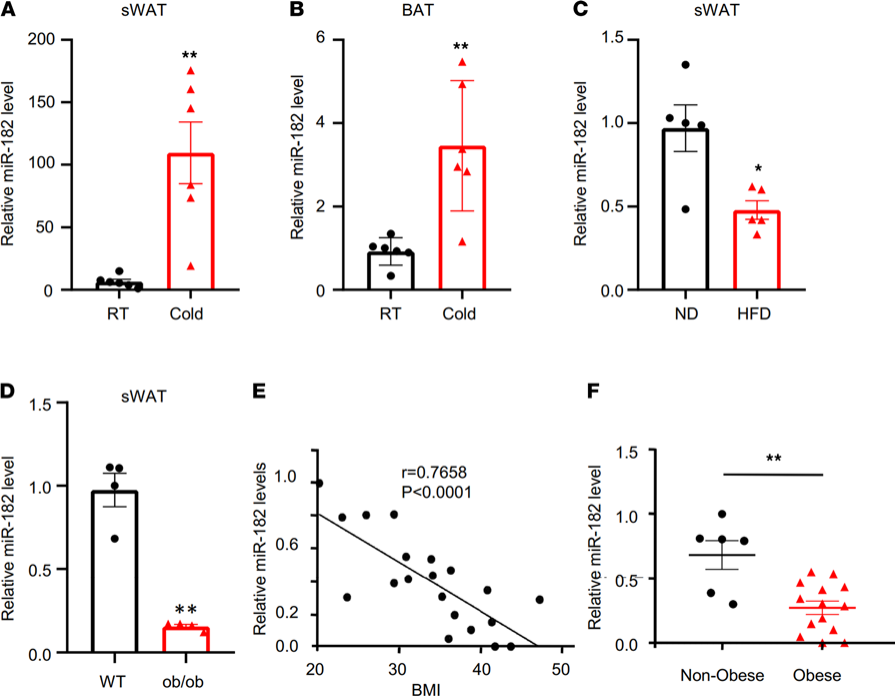
Adipose miR-182-5p expression is negatively correlated with obesity in mice and humans and is stimulated by cold exposure in mice. (**A** and **B**) The relative levels of miR-182-5p in sWAT or BAT of C57BL/6 mice maintained at 25°C (room temperature, RT) or exposed to 4°C for 1 week. (*n* = 6/group.) (**C**) Expression levels of miR-182-5p in sWAT of C57BL/6 mice fed with HFD or normal diet for 16 weeks (*n* = 5/group). (**D**) Expression levels of miR-182-5p in sWAT of *ob/ob* and WT mice (*n* = 4/group). (**E**) Regression analysis of miR-182-5p expression data from sWAT of human subjects with different BMIs. (**F**) Relative miR-182-5p expression from subcutaneous fat from nonobese (BMI < 30) and obese (BMI ≥ 30) human subjects. Data represent mean ± SEM. Significance determined by unpaired 2-tailed Student’s *t* test. *P < 0.05; **P < 0.01.

To determine the potential role of miR-182-5p in the beiging process, we exposed mice to cold. We found that cold exposure greatly increased miR-182-5p levels in both subcutaneous WAT (sWAT) (Figure 1A) and BAT (Figure 1B) of mice. Interestingly, miR-182-5p had a much greater response to cold exposure in sWAT (~100-fold change) than in BAT (~3-fold change), suggesting that miR-182-5p may have a major function in regulating beige fat thermogenesis. miR-182-5p expression levels were significantly reduced in sWAT of HFD-fed mice (Figure 1C) and *ob/ob* mice (Figure 1D) compared with their respective control mice. The expression of miR-182-5p in sWAT was also negatively correlated with BMI in humans (Figure 1, E and F). These data suggest that miR-182-5p downregulation may contribute to diet-induced obesity.

### miR-182-5p promotes beiging of WAT and energy expenditure in mice.

**Figure 2:**
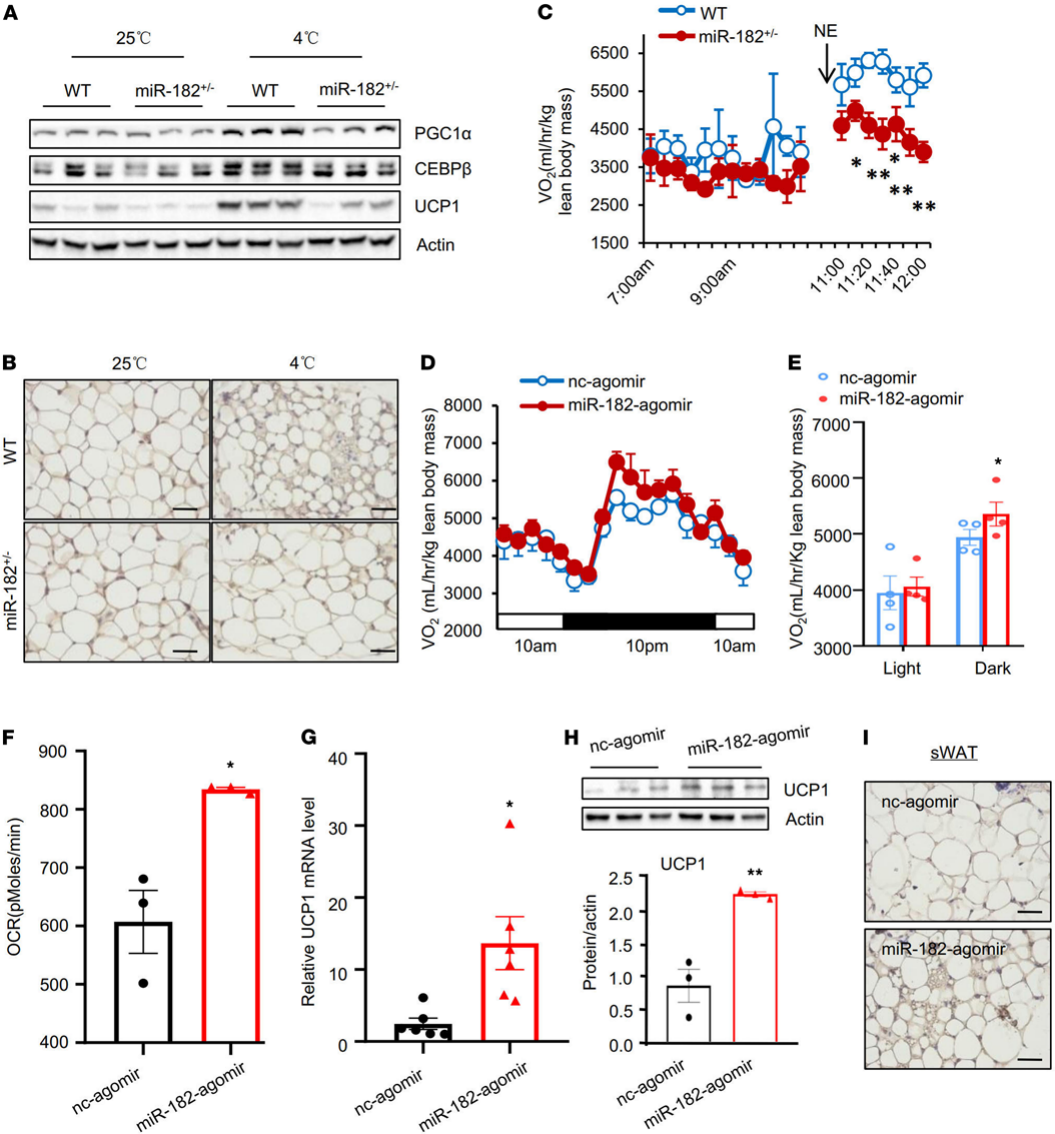
miR-182-5p is a key regulator of thermogenic gene expression and energy expenditure in mice. Male miR-182-5p heterozygous knockout mice (miR-182-5p^+/–^) and their WT littermates (8 weeks old) were housed individually in cages and exposed to cold (4°C; 6 h/d) for 7 days. (**A**) The protein levels of PGC1α, C/EBPβ, and UCP1 in sWAT were determined by Western blot (*n* = 3/group). (**B**) Immunohistochemical staining of UCP1 in sWAT of the miR-182-5p^+/–^ mice and WT control mice (*n* = 3/group; scale bar: 100 μm). (**C**) miR-182-5p^+/–^ mice and WT control mice (*n* = 5–7/group) (8 weeks old) were given an intraperitoneal injection of 1 mg/kg norepinephrine (NE) 1 hour, and O_2_ consumption was measured by Comprehensive Lab Animal Monitoring System (CLAMS) after NE injection. (**D**) O_2_ consumptions of miR-182-5p agomir- or control agomir-injected (fat pad) mice (*n* = 4/group) (8 weeks old) were measured by indirect calorimetry using CLAMS. (**E**) Mean O_2_ consumption was normalized to lean body mass and analyzed by Student’s *t* test. (**F**) OCR of sWAT of miR-182-5p agomir- or negative control (nc) agomir-injected (fat pad) mice (*n* = 3/group). Volume O_2_ consumption was normalized to 10 g tissue weight. (**G**) The relative mRNA level of Ucp1 in the miR-182-5p agomir- or nc agomir-injected fat pad were determined by quantitative real-time PCR (qRT-PCR) (*n* = 6/group). (**H**) The expression of UCP1 protein in mouse fat pad injected with miR-182-5p agomir- or negative control (nc) agomir was determined by Western blot (*n* = 3/group). (**I**) Immunohistochemical staining of UCP1 in sWAT of the miR-182-5p agomir- or nc agomir-injected (fat pad) mice (*n* = 3/group; scale bar: 100 μm). Data represent mean ± SEM. Significance determined by unpaired 2-tailed Student’s *t* test. *P < 0.05; **P < 0.01.

Next, we investigated whether miR-182-5p deficiency affects cold-induced thermogenic gene expression in mice. Because some of the homozygous miR-182-5p–knockout mice were born with defects, such as shorter tails and lack of ears, which may complicate the interpretation of the data, we used heterozygous miR-182-5p–knockout mice (miR-182-5p^+/–^) in our study, which showed a significant decrease in miR-182-5p levels in both sWAT (Supplemental Figure 1A; supplemental material available online with this article; https://doi.org/10.1172/jci.insight.150249DS1) and BAT (Supplemental Figure 1B) compared with their control mice but did not show any detectable growth, morphological, and metabolic defects under normal housing conditions. While miR-182-5p deficiency in mice had no effect on cold exposure–induced uncoupling protein 1 (*Ucp1*) gene expression in BAT (Supplemental Figure 1C), it greatly suppressed cold-induced protein expression of key regulators of beige fat thermogenesis, such as UCP1, PPARγ coactivator 1α (PGC1α), and C/EBPβ, in sWAT (Figure 2A), as well as the formation of multilocular brown-like adipocytes (Figure 2B). Consistent with these results, miR-182-5p^+/–^ mice displayed a significant reduction in norepinephrine-induced (NE-induced) oxygen consumption (Figure 2C) and impaired cold tolerance at 4°C (Supplemental Figure 1D) compared with control littermates. In addition, the miR-182-5p^+/–^ mice gained more body weight on HFD at room temperature (Supplemental Figure 1E), probably due to impaired beige fat thermogenesis. Taken together, these results suggest that miRNA-182-5p is an important regulator of energy expenditure.

To further determine the potential role of adipose miR-182-5p in regulating beige fat development and thermogenesis in vivo, we established a fat pad injection technique using a modified procedure as described (13, 14). Subcutaneous fat pad injection of miR-182-5p agomir, a synthetic double-stranded miRNA that mimics endogenous miR-182-5p, significantly increased miR-182-5p level in sWAT (Supplemental Figure 1F) but not in BAT and epididymal WAT (eWAT) (Supplemental Figure 1, G and H), demonstrating the specificity of the injection. The miR-182-5p agomir-treated mice had higher oxygen consumption compared with nonspecific agomir-treated control mice at room temperature (Figure 2, D and E). Furthermore, bioenergetics analysis using a Seahorse Extracellular Flux Analyzer revealed that basal oxygen consumption rate (OCR) was significantly increased in sWAT of the miR-182-5p agomir-treated mice compared with nonspecific agomir-treated control mice (Figure 2F). In agreement with these findings, the mRNA (Figure 2G) and protein (Figure 2H) levels of UCP1, as well as the formation of multilocular brown-like adipocytes (Figure 2I), were greatly increased in sWAT but not in BAT and eWAT (Supplemental Figure 1, I and J) of the miR-182-5p agomir-treated mice. Together, these results demonstrated that miR-182-5p is sufficient to promote beiging of sWAT and energy expenditure in vivo.

### miR-182-5p promotes white adipose beiging and thermogenesis via a macrophage-dependent mechanism.

**Figure 3:**
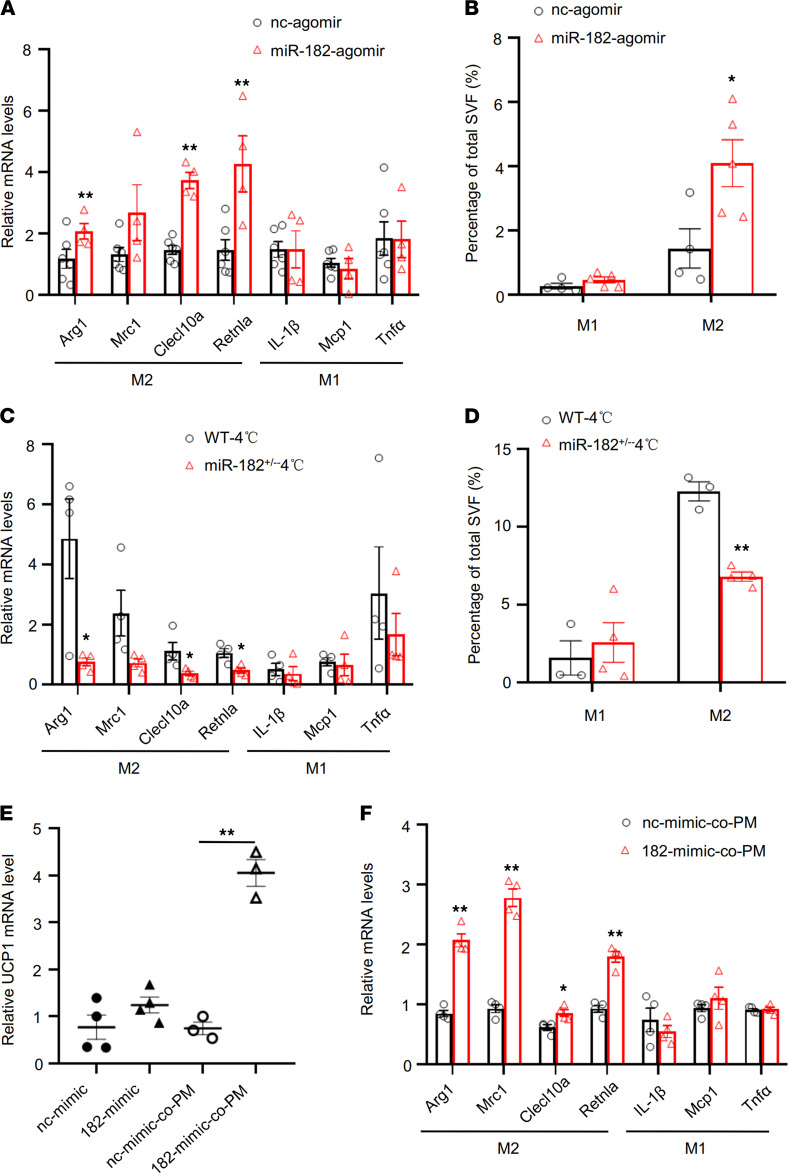
miR-182-5p promotes thermogenic gene expression in adipocytes via a macrophage-dependent mechanism. (**A**) qRT-PCR analysis of mRNA levels of M1 and M2 marker genes in sWAT of miR-182-5p agomir- or control agomir-injected (fat pad) mice (*n* = 4–6/group). (**B**) Flow cytometry analysis showing the percentage of total stromal vascular fractions (SVFs), and M1 and M2 macrophages in sWAT of miR-182-5p agomir- or control agomir-injected (fat pad) mice (*n* = 4–5/group). qRT-PCR analysis for mRNA levels of M1 and M2 marker genes (**C**) and flow cytometry analysis showing the percentage of M1 and M2 macrophages in SVFs (**D**) from sWAT of miR-182-5p^+/–^ and WT control mice under cold exposure conditions (*n* = 3–4/group). miR-182-5p mimic (182-mimic) or its negative control (nc-mimic) were overexpressed in mouse primary adipocytes. The cells were cultured alone or cocultured with mouse peritoneal macrophages (PM) for 3 days (*n* = 3–4/group). (**E**) The Ucp1 mRNA levels were determined in mouse primary adipocytes by qRT-PCR. (**F**) qRT-PCR analysis for mRNA levels of M1 and M2 marker genes. Data represent mean ± SEM. Significance determined by unpaired 2-tailed Student’s *t* test (**A**–**D** and **F**) and by 1-way ANOVA (**E**). *P < 0.05; **P < 0.01.

To determine the cellular mechanism by which miR-182-5p promotes beiging of white adipocytes and thermogenic gene expression, we overexpressed miR-182-5p in sWAT-derived primary adipocytes. To our surprise, overexpression of the miR-182-5p mimic did not promote beiging and thermogenic genes’ expression in primary white adipocytes cultured alone (Supplemental Figure 2, A and B), suggesting that the in vivo effect of miR-182-5p on white adipose beiging and thermogenesis may depend on the ATME regulated by crosstalk between adipocytes and their neighboring cells in adipose tissue. We tested several markers of T cells, eosinophils, and macrophages in sWAT of miR-182-5p agomir-injected mice. No significant difference in the markers of T cells (*Tbet*, *Ifn-*γ, *Gata3*, and *Foxp3*) and eosinophils (*SiglecF*) was observed between mice treated with or without miR-182-5p agomir (Supplemental Figure 2C). However, we found that the expression levels of M2 macrophage markers, such as arginase 1 (*Arg1*), *Clecl10a*, and *Retnla*, were all significantly upregulated in sWAT of mice treated with miR-182-5p agomir via fat pad injection (Figure 3A), which was concurrent with increased UCP1 expression (Figure 2, G and H). Increased M2 macrophage accumulation was also observed by F4/80 + CD206 sorting in sWAT from miR-182-5p agomir-treated mice compared with control mice (Figure 3B). On the other hand, suppressing miR-182-5p expression significantly decreased cold-induced expression of M2 macrophage–selective genes (Figure 3C) and M2 macrophage accumulation (Figure 3D) in sWAT of mice, suggesting that macrophages were involved in miR-182-5p–induced beiging of WAT. To determine whether macrophages are involved in miR-182-5p–induced beiging fat thermogenesis, we overexpressed miR-182-5p in primary white adipocytes and cocultured the cells with macrophages. Overexpression of miR-182-5p had no effect on UCP1 mRNA expression in primary white adipocytes cultured alone (Supplemental Figure 2, A and B) but significantly increased UCP1 mRNA levels in primary white adipocytes cocultured with peritoneal macrophages (Figure 3E). In addition, miR-182-5p in adipocytes promoted the expression of M2 macrophage–selective genes, such as *Arg1*, *Mrc1*, *Clecl10a*, and *Retnla* (Figure 3F). Increased UCP1 mRNA expression was also detected in miR-182-5p mimic-treated adipocytes cocultured with bone marrow–derived macrophages (BMDMs) (Supplemental Figure 2D). On the other hand, overexpression of the miR-182-5p mimic in peritoneal macrophages did not stimulate UCP1 expression in cocultured white adipocytes (Supplemental Figure 2E), suggesting miR-182-5p mainly functions in white adipocytes rather than in macrophages to regulate the thermogenic crosstalk between these 2 cell types. Together, these results indicated that miR-182-5p promotes beiging of white adipocytes via an M2 macrophage–dependent mechanism.

### FGF21 mediates the promoting effect of miR-182-5p on beiging via a macrophage-dependent paracrine mechanism.

**Figure 4:**
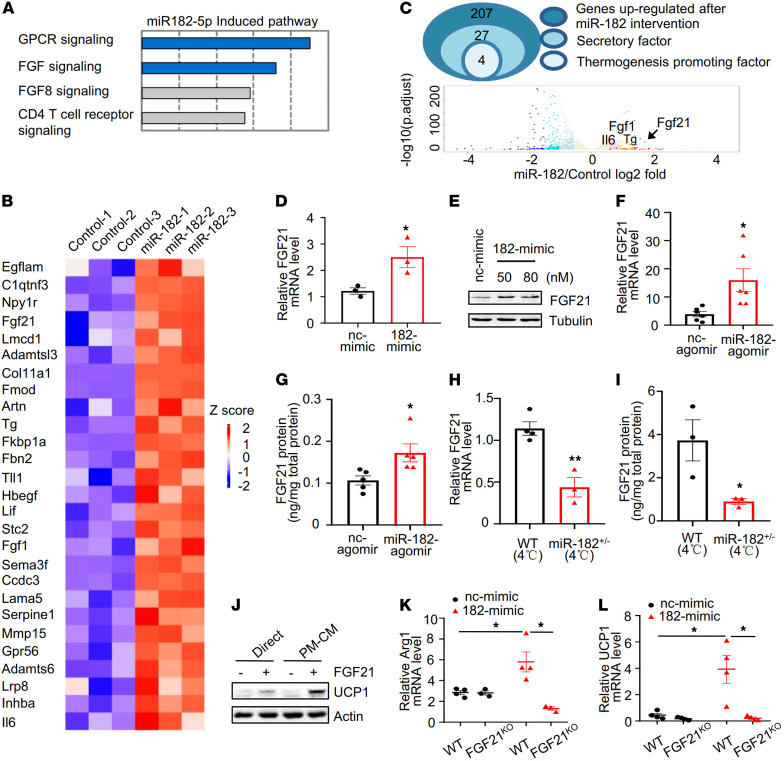
FGF21 mediates the promotive effect of miR-182-5p on UCP1 expression in adipocytes via a macrophage-dependent mechanism. (**A**) INOH pathway analysis of the RNA-Seq data from primary adipocytes overexpressing miR-182-5p (*n* = 3/group). (**B**) Heatmap analysis shows the relative expression levels of secretory genes. (**C**) The Venn diagram showing the strategy to identify thermogenesis-related secretory factors. Volcano plot depicting the gene expression differences between miR-182-5p intervention and control group. Upregulated genes were colored in red, and downregulated genes were colored in blue. (**D**) FGF21 mRNA and (**E**) protein levels in primary adipocytes overexpressing miR-182-5p mimic (182-mimic) or its negative control (nc-mimic) were determined by qRT-PCR (*n* = 3/group) or Western blot, respectively. (**F**) FGF21 mRNA and (**G**) protein levels in sWAT of miR-182-5p agomir- or nc-agomir–injected (fat pad) mice (*n* = 5–6/group) were determined by qRT-PCR or ELISA, respectively. (**H**) qRT-PCR analysis for FGF21 mRNA levels and (**I**) ELISA analysis for FGF21 protein concentration in sWAT of miR-182-5p^+/–^and WT control mice exposed to cold (*n* = 3–4/group). (**J**) PMs were treated with or without FGF21 (50 nM) for 48 hours, and conditioned medium (CM) of the cells was collected. Primary adipocytes were incubated with the FGF21-treated (+) or nontreated (-) CM or directly treated with FGF21 for 3 days. UCP1 expression in cells was determined by Western blot analysis (*n* = 3 biological replicates). 182-mimic or nc-mimic was overexpressed in primary white adipocytes isolated from FGF21^KO^ and their control mice. The cells were cocultured with PMs for 3 days, and the mRNA levels of (**K**) Arg1 and (**L**) UCP1 were determined by qRT-PCR (*n* = 4/group). Data represent mean ± SEM. Significance determined by unpaired 2-tailed Student’s *t* test (**D** and **F**–**I**) and by 1-way ANOVA (**K** and **L**). *P < 0.05; **P < 0.01.

Several cytokines, such as IL-4, IL-13, and IL-33, have been reported to stimulate alternative activation of macrophages and mediate cold-induced beige fat thermogenesis (3, 15–18). However, we found that miR-182-5p agomir treatment had no significant effect on the mRNA levels of these cytokines in vivo (Supplemental Figure 3A), suggesting the presence of a potentially novel mechanism by which miR-182-5p promotes thermogenic gene expression in primary white adipocytes. To test the underlying mechanism of miR-182-5p–mediated crosstalk between adipocytes and macrophages, we profiled mRNA expression by RNA-Seq analysis in mouse primary white adipocytes overexpressing miR-182-5p mimic or its mimic control. We have identified approximately 857 genes that are either upregulated or downregulated using 1.5-fold difference and adjusted *P* value (false positive rate) less than 0.05 as the cutoff criteria. INOH pathway analysis by InnateDB revealed a significant correlation between miR-182-5p and several biology processes, such as FGF signaling and the GPCR pathway (Figure 4A). We further analyzed the upregulated genes using UniProt and Gene Ontology term extracellular matrix and identified 27 genes encoding secreted proteins that were significantly increased in adipocytes overexpressing the miR-182-5p mimic compared with the controls (Figure 4B). GeneCard and PubMed database analysis revealed 4 genes that encode thermogenesis-interrelated secretory factors, including FGF21, acidic fibroblast growth factor (FGF1), interleukin-6 (IL6), and thyroglobulin (Tg) (Figure 4C). Volcano plot revealed that FGF21 was the most significantly differentially expressed gene between cells treated with or without miR-182-5p mimic (Figure 4C). Consistent with these findings, the mRNA (Figure 4D) and protein (Figure 4E) levels of FGF21 were both significantly increased in adipocytes overexpressing the miR-182-5p mimic compared with respective controls. The mRNA (Figure 4F) and protein (Figure 4G) levels of FGF21 were also significantly increased in sWAT of miR-182-5p agomir-treated mice compared with their control mice. On the other hand, suppressing miR-182-5p expression reduced the promoting effect of cold exposure on FGF21 expression in mouse sWAT (Figure 4, H and I). Taken together, these results demonstrate an important role of miR-182-5p in promoting adipocyte FGF21 expression in cell culture and in vivo.

To further investigate the mechanism by which FGF21 induces beige fat thermogenesis, we treated primary white adipocytes with FGF21 or incubated them with the conditioned medium (CM) from FGF21-treated mouse peritoneal macrophages. In agreement with a previous finding (6), FGF21 treatment directly stimulated UCP1 expression in primary adipocytes (Figure 4J). However, a much greater stimulatory effect of FGF21 on UCP1 expression was observed when adipocytes were incubated with CM from FGF21-treated macrophages (Figure 4J). Consistent with these results, incubating adipocytes with CM from FGF21-treated BMDMs significantly stimulated the mRNA levels of UCP1 in adipocytes, which was associated with increased mRNA levels of macrophage *Arg1*, a marker of M2 macrophage polarization (Supplemental Figure 3, B and C). Together, these results suggest that FGF21 induces beige fat thermogenesis mainly through a macrophage-dependent mechanism.

To define the role of FGF21 in miR-182-5p–induced beige fat thermogenesis, we overexpressed miR-182-5p mimic or its negative control in primary white adipocytes isolated from WT or FGF21-knockout mice. We found that coculturing miR-182-5p–overexpressed WT primary adipocytes with peritoneal macrophages significantly increased *Arg1* expression in macrophages, but the stimulatory effect of miR-182-5p on *Arg1* expression was significantly diminished in macrophages cocultured with primary white adipocytes isolated from FGF21-knockout mice (Figure 4K). Consistent with this result, miR-182-5p–induced UCP1 mRNA expression was blocked in primary white adipocytes isolated from FGF21-knockout mice cocultured with peritoneal macrophages (Figure 4L). Taken together, these findings strongly suggest that miR-182-5p promotes beige fat thermogenesis via a FGF21-stimulated and macrophage-dependent paracrine mechanism.

How does miR-182-5p increase the expression of FGF21? Bioinformatic analysis predicted that miR-182-5p could bind to the mRNA of Nr1d1 (also named Rev-Erbα) at 5′-UTR sites between 543 and 550 (Supplemental Figure 4A). This finding is interesting given that Nr1d1 has been shown to suppress FGF21 expression (19). Indeed, Nr1d1 mRNA levels were significantly decreased in sWAT of miR-182-5p agomir-treated mice (Supplemental Figure 4B) and in primary white adipocytes overexpressing miR-182-5p (Supplemental Figure 4C). On the other hand, Nr1d1 mRNA levels were significantly increased in sWAT of miR-182-5p^+/–^ mice compared with control mice (Supplemental Figure 4D) and in miR-182-5p–knockdown primary white adipocytes (Supplemental Figure 4E). Consistent with the finding that cold exposure increased miR-182-5p expression (Figure 1A), Nr1d1 mRNA levels were significantly decreased in sWAT of cold-exposed C57BL/6 mice (Supplemental Figure 4F). Importantly, luciferase reporter assay showed that the miR-182-5p mimic reduced the activity of the reporter with a WT Nr1d1 5′-UTR but not the one with mutations in the seed sequence (Supplemental Figure 4G), revealing that miR-182-5p inhibited Nr1d1 expression by directly interacting with the predicted target sites at 5′-UTR. Consistently, adenovirus-mediated overexpression of Nr1d1 greatly inhibited FGF21 protein expression in primary white adipocytes (Supplemental Figure 4H). Conversely, knockdown of Nr1d1 increased FGF21 and UCP1 levels in primary white adipocytes cocultured with peritoneal macrophages (Supplemental Figure 4I). Taken together, these results demonstrate that miR-182-5p enhances FGF21/macrophage axis–mediated beige thermogenesis by inhibiting Nr1d1 expression in white adipocytes. Nr1d1 is a key circadian clock component (Supplemental Figure 4J) that is directly regulated by brain and muscle ARNT-like 1 (20) and mediates the circadian patterning of cold tolerance (21). However, we found that the expression of miR-182-5p was not affected by circadian regulation (Supplemental Figure 4K). Nevertheless, further studies will be needed to determine whether or not miR-182-5p plays a role in circadian regulation of thermogenesis via modulating Nr1d1 expression.

### The miR-182-5p/FGF21 axis promotes beige thermogenesis via macrophage-dependent activation of the nAChR/PKA signaling.

**Figure 5:**
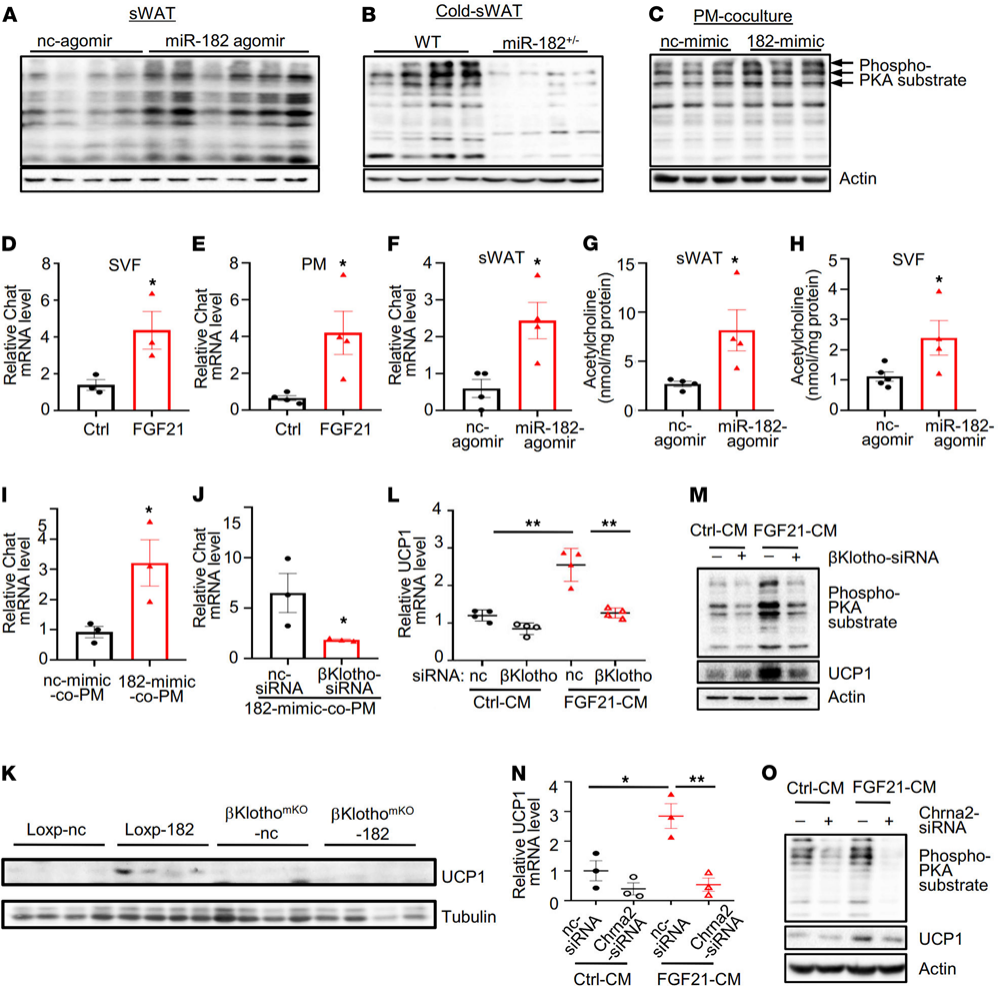
miR-182-5p promotes PKA activation via the nAChR signaling pathway. PKA substrate phosphorylation (**A**) in sWAT of mice injected with miR-182-5p agomir or control agomir, (**B**) in sWAT of miR-182-5p^+/–^ and WT control mice, and (**C**) in miR-182-5p–overexpressed primary adipocytes cocultured with or without PMs (*n* = 3/group). Choline acetyltransferase (Chat) mRNA levels were determined by qRT-PCR in (**D**) SVFs and (**E**) PM treated with or without FGF21 (*n* = 3–4/group). (**F**) Chat mRNA levels in sWAT of mice injected with miR-182-5p agomir- or control agomir. ELISA analysis of acetylcholine levels secreted from the (**G**) sWAT and (**H**) inguinal SVF of mice injected with miR-182-5p agomir or control agomir (*n* = 4/group). (**I**) Chat mRNA levels in PMs cocultured with primary white adipocytes overexpressing 182-mimic or nc-mimic were determined by qRT-PCR (*n* = 3/group). (**J**) Chat mRNA levels in βKlotho-siRNA or nc-siRNA–treated PMs cocultured with primary white adipocytes overexpressing miR-182-5p mimic were determined by qRT-PCR (*n* = 3/group). (**K**) UCP1 protein levels in sWAT of βKlotho^mKO^ mice and *Loxp* control mice injected with miR-182-5p agomir (-182) or control agomir (-nc) (*n* = 4/group). The mRNA (**L**) and protein (**M**) levels of UCP1 or PKA substrate phosphorylation (**M**) in primary adipocytes incubated with CM from control (nc-siRNA) or βKlotho-suppressed PMs treated with or without FGF21 were determined by qRT-PCR or Western blot (*n* = 3 biological replicates). The mRNA (**N**) and protein (**O**) levels of UCP1 or PKA substrate phosphorylation (**O**) in Chrna2-siRNA– or nc-siRNA–treated primary adipocytes incubated with CM from FGF21-treated or nontreated PMs were determined by qRT-PCR or Western blot (*n* = 3 biological replicates). Data represent mean ± SEM. Significance determined by unpaired 2-tailed Student’s *t* test (**D**–**J**) and by 1-way ANOVA (**L** and **N**). *P < 0.05; **P < 0.01.

To determine the potential mechanism by which the miR-182-5p/FGF21 axis promotes white adipose beiging and thermogenesis, we first examined the phosphorylation of PKA substrates in sWAT of mice treated with miR-182-5p agomir. PKA substrate phosphorylation was markedly increased in sWAT of miR-182-5p agomir-treated mice compared with their control mice (Figure 5A). On the other hand, cold exposure–induced PKA substrate phosphorylation was greatly suppressed in sWAT of miR-182-5p^+/–^ mice compared with their control mice (Figure 5B), suggesting that miR-182-5p plays a key role in regulating activation of the PKA signaling in sWAT of mice. In addition, the stimulatory effect of miR-182-5p on the phosphorylation of PKA substrates could not be detected in primary white adipocytes cultured alone (Supplemental Figure 5A) but could be observed in primary white adipocytes cocultured with peritoneal macrophages (Figure 5C), suggesting that miR-182-5p promotes beige adipocytes’ thermogenesis via macrophage-mediated PKA activation. There is some evidence suggesting that M2 macrophages stimulate PKA activation and thermogenic gene expression in adipocytes by promoting NE release from M2 macrophages (15, 16, 22). However, we found no obvious changes in Th protein levels and/or NE levels in either miR-182-5p–overexpressed mice or miR-182-5p^+/–^ mice (Supplemental Figure 5, B–E), suggesting the presence of a potentially novel mechanism by which macrophages promote activation of the PKA signaling in adipocytes.

A recent study showed that beige adipocyte thermogenesis could be stimulated by the nAChR signaling pathway, which is activated by acetylcholine locally produced from M2 macrophages of adipose tissue–resident SVFs (23). To determine the role of M2 macrophages in regulating thermogenic gene expression in white adipocytes, we treated SVFs isolated from C57BL/6 mice with or without FGF21. As expected, FGF21 treatment increased total and M2-polarized macrophages in the SVFs (Supplemental Figure 5F). Interestingly, treating sWAT-derived SVFs or peritoneal macrophages with FGF21 significantly increased the mRNA levels of Chat (Figure 5, D and E), the key enzyme involved in acetylcholine synthesis. Consistent with this result, a significant increase in Chat expression (Figure 5F) and acetylcholine production (Figure 5G) was also observed in sWAT of miR-182-5p agomir-injected mice. Next, we examined acetylcholine production in SVFs isolated from sWAT of miR-182-5p agomir-treated mice. A significant increase in acetylcholine levels was detected in SVFs isolated from sWAT of miR-182-5p agomir-treated mice compared with their control mice (Figure 5H). Chat expression was also significantly upregulated in peritoneal macrophages cocultured with the miR-182-5p mimic-treated primary white adipocytes (Figure 5I). A similar result was obtained in BMDMs cocultured with primary white adipocytes overexpressing miR-182-5p (Supplemental Figure 5G). To provide further evidence on the role of the miR-182-5p/FGF21 axis in promoting acetylcholine production in macrophages, we overexpressed miR-182-5p in primary white adipocytes, which were cocultured with peritoneal macrophages treated with or without βKlotho-siRNA (Supplemental Figure 5H), the essential coreceptor to activate FGF21 signaling. Suppressing βKlotho in peritoneal macrophages greatly inhibited the miR-182-5p/FGF21 axis–induced expression of Chat (Figure 5J), further demonstrating a key role of the miR-182-5p/FGF21 axis in promoting Chat expression and acetylcholine production in macrophages. To provide further evidence on the role of miR-182-5p/FGF21 axis in promoting beige adipocytes’ thermogenesis via a macrophage acetylcholine-dependent paracrine mechanism, we microinjected miR-182-5p agomir to the subcutaneous fat pad of macrophage-specific βKlotho (the coreceptor of FGF21 receptor) knockout (βKlotho^mKO^) mice and their *loxp* control mice. Suppressing βKlotho in macrophages greatly inhibited the miR-182-5p-induced expression of UCP1, which is associated with reduced Chat expression in sWAT of mice (Figure 5K). Furthermore, we found that treating primary white adipocytes with CM from FGF21-stimulated peritoneal macrophages significantly increased the mRNA and protein expression of UCP1 as well as the phosphorylation of PKA substrates, but the stimulatory effect of CM was greatly reduced when βKlotho was suppressed by siRNA in the peritoneal macrophages (Figure 5, L and M).

To determine if the promoting effect of the miR-182-5p/FGF21 axis on thermogenic gene expression is acetylcholine signaling dependent, we suppressed the expression of the nAChR by Chrna2-siRNA in mouse primary white adipocytes, which were cultured with CM from control- or FGF21-treated peritoneal macrophages. As expected, treating primary white adipocytes with CM from FGF21-stimulated macrophages significantly increased the expression of UCP1 (Figure 5, N and O). Knockdown of nAChR by Chrna2-siRNA in primary white adipocytes (Supplemental Figure 5I) significantly reduced FGF21-CM–stimulated UCP1 mRNA (Figure 5N) and protein (Figure 5O) levels as well as the phosphorylation of PKA substrates (Figure 5O), which are known to be activated by the nAChR signaling pathway to stimulate the thermogenic program. Taken together, these findings suggest that the adipose miR-182-5p/FGF21 axis promotes PKA activation via a paracrine loop, which requires macrophage acetylcholine production and adipocyte nAChR signaling.

### miR-182-5p overexpression in subcutaneous fat pad of mice resists obesity and its metabolic consequences.

**Figure 6:**
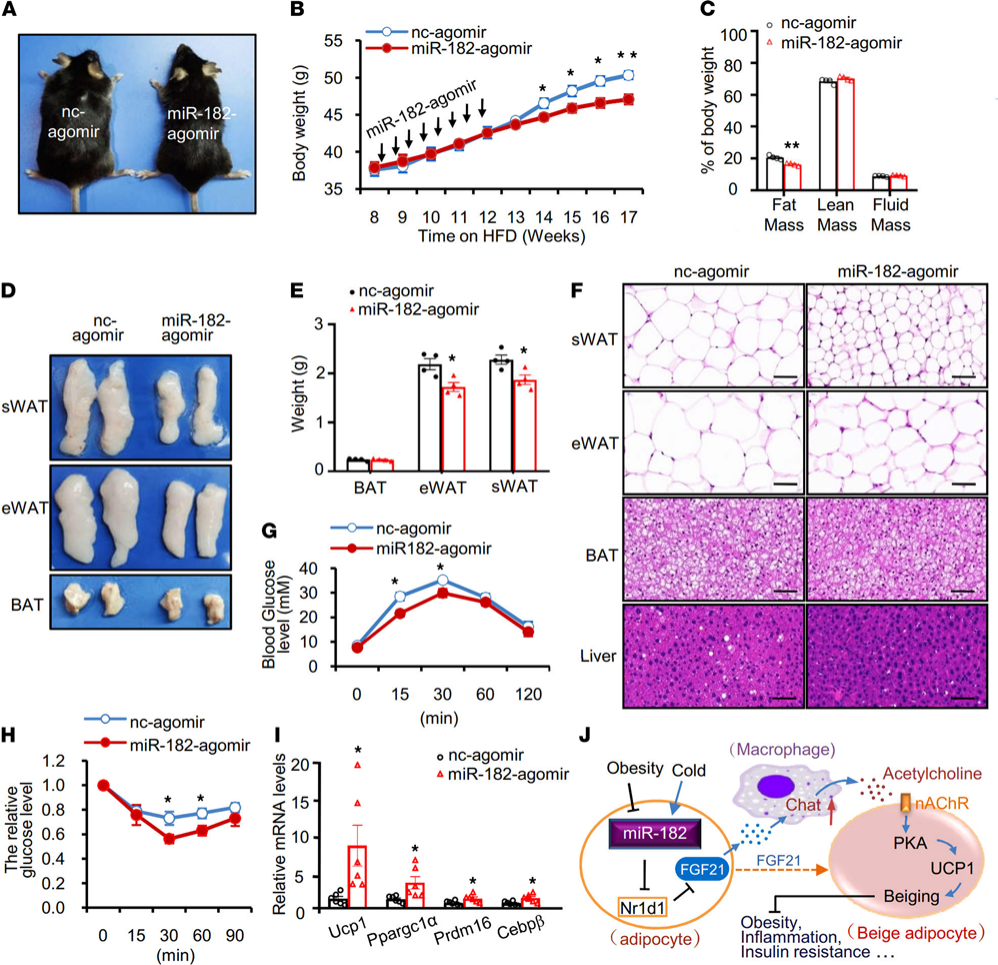
miR-182-5p overexpression in subcutaneous fat pad of mice resists obesity and its metabolic consequences. C57BL/6 mice were fed HFD for 8 weeks and then received subcutaneous fat pad injection of miR-182-5p agomir or control agomir (nc-agomir) every 3 days for 8 injections. (**A**) Representative images of miR-182-5p agomir and nc-agomir mice after a 17-week HFD feeding. (**B**) Body weight (BW) of miR-182-5p agomir and nc-agomir mice fed with HFD for 17 weeks (*n* = 4/group). (**C**) Body composition of HFD-fed miR-182-5p agomir and nc-agomir mice (*n* = 4/group). (**D**) Representative images of fat pads from HFD-fed miR-182-5p agomir and nc-agomir mice. (**E**) Weights of sWAT, eWAT, and BAT of HFD-fed miR-182-5p agomir and nc-agomir mice (*n* = 4/group). (**F**) Representative images of hematoxylin and eosin staining of sWAT, eWAT, BAT, and liver sections from HFD-fed miR-182-5p agomir and nc-agomir mice (*n* = 3 biological replicates; scale bar: 100 μm). Glucose tolerance tests (**G**) and insulin tolerance tests (**H**) were performed in HFD-fed miR-182-5p agomir and nc-agomir mice (*n* = 4–5/group) according to similar procedures as described in our previous studies (32). (**I**) mRNA levels of thermogenic marker genes in sWAT of HFD-fed miR-182-5p agomir and nc-agomir mice were quantified by qRT-PCR and normalized to β-actin (*n* = 6/group). (**J**) A proposed model of the mechanism by which miR-182-5p promotes thermogenic gene expression in white adipocytes. Data represent mean ± SEM. Significance determined by unpaired 2-tailed Student’s *t* test. *P < 0.05; **P < 0.01.

To determine whether miR-182-5p overexpression–induced beiging of WAT could counteract overnutrition-induced obesity and its associated metabolic syndrome, we injected miR-182-5p agomir or control agomir into mice that had been fed HFD for 8 weeks every 3 days for 8 injections. miR-182-5p agomir injection into mice had no significant effect on food intake (data not shown) but greatly reduced body weight (Figure 6, A and B) and fat mass (Figure 6C). In agreement with these findings, the size (Figure 6D) and weight (Figure 6E) of sWAT and eWAT were both significantly decreased in miR-182-5p agomir-treated mice compared with their control mice, whereas BAT weight was unaltered (Figure 6E). In addition, the cell sizes of white adipocytes were generally smaller in the miR-182-5p agomir-treated mice than in the control mice (Figure 6F). miR-182-5p agomir treatment also greatly ameliorated HFD-induced liver steatosis (Figure 6F), glucose intolerance (Figure 6G), and insulin resistance (Figure 6H) in mice. Consistently, the expression of beige fat thermogenesis markers, such as *Ucp1*, *Ppargc1*α, *Prdm16*, and *Cebp*β, were all increased in the sWAT (Figure 6I) but not in the BAT (Supplemental Figure 6A) of miR-182-5p agomir-treated mice. These results strongly indicate that miR-182-5p overexpression in subcutaneous fat pad promotes beiging of WAT and resists diet-induced obesity.

## Discussion

Increasing beige fat thermogenesis has a potential beneficial effect against obesity and obesity-associated diseases. In the current study, we uncover a novel miR-182-5p/FGF21/acetylcholine/PKA axis that plays a critical role in cold-induced beige fat thermogenesis. Mechanistically, miR-182-5p promotes FGF21 expression and secretion by suppressing Nr1d1 in white adipocytes, leading to increased expression and secretion of acetylcholine in macrophages that activates PKA signaling and thermogenesis in beige adipocytes (Figure 6J). Our findings reveal an important ATME crosstalk between macrophages and adipocytes that plays a critical role in regulating beige fat thermogenesis.

miR-182-5p has been shown to play a role in regulating glucose homeostasis in skeletal muscle (24). However, its role in regulating white adipose beiging progress and thermogenesis was unknown. We found that a 35%–40% reduction in miR-182-5p levels in sWAT was sufficient to reduce cold-induced UCP1 expression in sWAT of mice (Figure 2, A and B), demonstrating miR-182-5p as a key regulator of beige fat thermogenesis and energy homeostasis. Interestingly, miR-182-5p deficiency had no effect on Ucp1 expression (Supplemental Figure 6, B and C) and macrophages’ polarization (Supplemental Figure 6D) in BAT of cold-exposed mice, indicating miR-182-5p plays a selective role in controlling thermogenic gene expression in beige fat but not in BAT. These results are consistent with the findings that both FGF21 (6) and the acetylcholine signaling pathway (25) have a major effect on beige but not BAT thermogenesis and that distinct mechanisms are present in WAT and BAT to regulate thermogenesis (23, 26, 27). How the tissue selectivity of miR-182-5p is established is unclear, but it is known that BAT has much lower numbers of macrophages compared with WAT (28, 29). Thus, it is possible that the great difference in macrophage numbers in BAT and sWAT contributes to the distinct responsiveness to miR-182-5p action by these tissues. It is also possible that the different microenvironment in BAT and sWAT has additional effects on the function of miR-182-5p and/or FGF21, thus resulting in a tissue-specific response to miR-182-5p–mediated signaling. Further experiments will be needed to address these questions.

miR-182-5p functions as a positive regulator of FGF21 in adipocytes, and upregulation of FGF21 plays a key role in mediating the crosstalk between adipocytes and macrophages in adipose tissue. This crosstalk generates an ATME that facilitates beige fat thermogenesis and energy expenditure, including acetylcholine release and alternative activation of macrophage. These results provide a mechanistic insight into the promotive roles of FGF21 in adaptive thermogenesis and its therapeutic potential in metabolic disorders. While it is well established that liver is the main site of serum FGF21 production to regulate glucose and lipid metabolism (30, 31), the functional roles and the mechanisms of action of adipose-derived FGF21 are much less clear. In adipose tissues, the promoting effect on beige fat thermogenesis is mainly contributed by locally produced FGF21 (6, 7). We show that FGF21 not only promotes alternative activation of macrophages but also induces acetylcholine production. Macrophage alternative activation has been known to be induced by FGF21; the stimulation of acetylcholine might be an important additional mechanism by which FGF21 induces beige fat thermogenesis through its paracrine function.

A recent study shows that cold induces the release of acetylcholine from adipose-derived SVFs, including M2 macrophages, which activates the nAChR signaling pathway in adipocytes and promotes beige fat thermogenesis (23). Interestingly, activation of this signaling pathway promotes thermogenic gene expression in sWAT but not BAT (23). However, the mechanism by which cold exposure activates the nAChR signaling pathway remained unknown. We found that cold exposure increased FGF21 expression in adipose tissue, concurrently with the increased Chat/Chrna2 expression in adipose tissue (Supplemental Figure 7, A and B). In addition, treating macrophages with FGF21 significantly increased the mRNA levels of Chat (Figure 5E), a key enzyme involved in the biosynthesis of acetylcholine. These findings reveal that cold-induced upregulation of FGF21 may activate beige fat thermogenesis by activation of the nAChR signaling pathway, uncovering a mechanism by which FGF21 regulates beige fat thermogenesis and energy homeostasis.

In conclusion, we have identified miR-182-5p as a key regulator that promotes beige fat thermogenesis. We show that miR-182-5p induces adipose FGF21 production by suppressing Nr1d1 expression, which in turn stimulates the macrophage-dependent acetylcholine/PKA axis in adipocytes to promote browning of white adipose tissues. Our study also identifies macrophage Chat as a target of adipose miR-182-5p/FGF21 and uncovers a potentially novel mechanism by which the ATME generated by the crosstalk between adipocytes and macrophages regulates beige fat thermogenesis.

## Methods

### Animals.

miR-182-5p heterozygous knockout mice (miR-182-5p^+/–^) were obtained from the Laboratory Animal Resource Bank, National Institute of Biomedical Innovation, Japan (https://animal.nibiohn.go.jp) (32). The βKlotho-floxed mice and FGF21-knockout mice were provided by Steven Kliewer (UT Southwestern Medical Center, Dallas, Texas, USA) and Aimin Xu (University of Hong Kong, Pokfulam, China), respectively. The *ob/ob* mice were from GemPharmatech Co., Ltd. Macrophage-specific βKlotho-knockout mice were generated by crossing βKlotho-floxed mice with Lysozyme-M Cre–transgenic mice (The Jackson Laboratory, 004781). For the HFD-induced obesity experiments, the mice were randomly distributed into weight-matched groups fed an HFD (60 kcal% fat, D12492; Research Diets Inc.). All animals were housed in a temperature-controlled environment with a 12-hour light/12-hour dark cycle and had access to food and water ad libitum.

### Fat pad injection.

miR-182-5p overexpression mice were established by a fat pad injection technique using a modified procedure as described (13, 14). Briefly, miR-182-5p agomir was injected into the inguinal fat pad of 8-week-old male mice, using a microsyringe equipped with a 31-gauge injection needle. For each mouse, miR-182-5p agomir (2.5 nmol, miR40000211-1-2, RIBOBIO; Exiqon) or its negative control (2.5 nmol, miR04201-1-10, RIBOBIO; Exiqon) was administered at 5 sites spread across the fat depot. At each site, the injection needle was inserted approximately 4 mm into the adipose tissue, and a 20 μL injection was made for approximately 30 seconds, with the needle held in place for at least 30 seconds to minimize reflux. The needle was withdrawn at approximately 2 mm, and a second 20 μL injection was made similarly to the initial injection. The injection was repeated every 3 days, and the mice were sacrificed after the sixth injection.

### Histological analysis.

For histological analysis, adipose tissues were fixed with a buffer containing 10% formalin for 24 hours and embedded in paraffin. For IHC, tissue sections (10 mm thick) were deparaffinized, rehydrated, and followed with antigen retrieval using heat-induced epitope methods as suggested by the antibody company (Abcam). The sections were then incubated with 1:800 diluted UCP1 antibody (MilliporeSigma, U6382), and IHC staining was performed using the HRP/DAB detection kit (Abcam).

### Immunoblot.

Cells or tissues were lysed in RIPA lysis buffer (P0013B, Beyotime) supplemented with protease inhibitor (4693159001, Roche) and phosphatase inhibitors (4906837001, Roche). Protein concentration was measured using a BCA protein assay kit (23225, Thermo Fisher Scientific). Proteins were separated by SDS-PAGE and transferred to nitrocellulose membranes using a Trans-Blot Turbo transfer system (Bio-Rad). Membranes were incubated for 1 hour at room temperature in blocking buffer (5% BSA, FA016, GENVIEW), then incubated at 4°C overnight with primary antibodies. Antibodies were incubated with horseradish peroxidase–conjugated secondary antibodies. The primary antibodies are described below. The secondary antibodies were from Promega (W4011, W4021). Signals were detected using ChemiDOC XRS+ and the Image Lab system (Bio-Rad).

### Energy expenditure.

Energy expenditure in mice was determined by metabolic cage (CLAMS) studies at either 25°C or standard room temperature to compare energy metabolism. Mean OCRs were normalized to lean body mass and analyzed by 2-tailed Student’s *t* test.

### Cold stress.

Male mice (8 weeks old) were individually housed in cages kept at room temperature (25°C) or cold temperature (4°C) under a 12-hour light/12-hour dark cycle. Food and water were available ad libitum. In cold temperature groups, mice were kept at 4°C for 7 days. Inguinal sWAT was isolated and subjected to further analyses.

### Human subjects.

Twenty patients (average age: 47.6 ± 3.55 years, 16 females and 4 males) were recruited from the Department of Surgery at the University of Texas Health at San Antonio (UTHSA, approved IRB protocol HSC20160323N). All subjects were undergoing abdominal plastic surgery. Preoperative BMI was calculated as BW in kilograms over squared height in meters. During the operation, abdominal sWATs (≈2 cc each) were collected, snap-frozen, and stored at −80°C before RNA extraction.

### Antibodies.

For Western blotting, we used anti-UCP1 (U6382, 1:1000) and anti–β-Actin (A3854, 1:40,000) from MilliporeSigma; anti-PGC1α (ab54481, 1:1000), anti-FGF21 (ab64857, 1:1000) from Abcam; anti-C/EBPβ (sc7962, 1:1000) from Santa Cruz Biotechnology; and anti-Nr1d1 (Rev-Erbα) (13418, 1:1000), anti–Tyrosine Hydroxylase (2792, 1:1000), and anti-Phospho-(Ser/Thr) PKA Substrate (9621, 1:1000) from Cell Signaling Technology. For flow cytometry, we used anti-F4/80 (123110, 1:200), anti-CD206 (141704, 1:200), anti-CD11b (101228, 1:200), and anti-CD11c (117310, 1:200) from BioLegend.

### Murine peritoneal macrophages.

We harvested the peritoneal macrophages by injecting and shaking 14 mL (7 mL × 2) of ice bath washing buffer containing RPMI-1640 (C11875500BT, Gibco, Thermo Fisher Scientific) and 1% heat-inactivated fetal bovine serum (FBS; 42Q6395K, Gibco, Thermo Fisher Scientific). Collected cells were centrifuged at 1250*g* for 3 minutes at 4°C. Cells were resuspended in 2 mL of culture medium and seeded in a culturing plate. To purify the macrophages, the cell culture medium was changed after seeding in a humidified atmosphere containing 5% CO_2_.

### Murine BMDMs.

BMDMs were prepared by flushing mouse femurs and tibias with RPMI-1640 medium (containing 100 U/mL of penicillin-streptomycin and 1% FBS). Cells were filtered through a 70 mm cell strainer and resuspended in complete RPMI-1640 medium (containing 100 U/mL of penicillin-streptomycin and 10% FBS). To generate BMDMs, cells were cultured for 7 days in complete medium containing 20 ng/mL of M-CSF (576406, BioLegend).

### Adipose stromal vascular cell isolation.

Mouse primary SVFs were isolated and cultured as described (32). Briefly, sWAT depots from 4-week-old male C57BL/6 mice were quickly isolated and minced. The tissue pieces were digested with type II collagenase (035M4034V, MilliporeSigma). The digested tissue was filtered through a 100 mm nylon screen. Collected cells were centrifuged at 1250*g* for 3 minutes at 4°C. Cells were resuspended in 2 mL of culture medium and seeded on a culture plate. Differentiation of cells was initiated according to the methods as described (33, 34).

### siRNA or miRNA mimic transfection and coculture assays.

siRNA (GenePharma) or miRNA mimic (Ambion) was transfected into the cells at a final concentration of 250 nmol/L (siRNA) or 50 nmol/L (miRNA-182-5p mimic) using the Lipofectamine RNAiMAX transfection reagent (13778075, Thermo Fisher Scientific), according to the manufacturer’s instructions. Adipocytes were cocultured with macrophages for 3 days using a Transwell plate (0.4 μm polycarbonate filter, Corning), with adipocytes placed in the lower chamber and macrophages in the upper chamber.

### RNA extraction and qRT-PCR.

Total RNAs were isolated and prepared using the TRIzol reagent (15596026, Thermo Fisher Scientific) following the manufacturer’s instructions. Reverse transcription of RNA was conducted using a RevertAid First Strand cDNA Synthesis Kit (0079095, Thermo Fisher Scientific). qRT-PCRs using the FastStart Universal SYBR Green Master (04913914001, Roche) were carried out on a 7900HT Fast Real-Time PCR System (Applied Biosystems, Thermo Fisher Scientific). The primer sequences for the genes tested were as follows, UCP1 forward: 5′-TACCAAGCTGTGCGATGTCCA-3′, reverse: 5′-GCACACAAACATGATGACGTTCC-3′. The relative levels of mRNA transcripts to control β-actin were determined by 2^-ΔΔCT^. The mature miRNA sequence for mouse miR-182-5p was UGUAAACAUCCUACACUCAGCU. The expression levels of miRNAs were normalized to that of U6 expression.

### RNA-Seq and pathway analysis.

Total RNA was isolated using TRIzol method and was processed in the Genomic Sequencing Facility in the Greeyhey Children’s Cancer Research Institute at UTHSA using next-generation sequencing on a HiSeq 3000 system (Illumina). Data were analyzed using the Database for Annotation, Visualization and Integrated Discovery (https://david.ncifcrf.gov/) and innateDB (https://www.innatedb.com).

### Luciferase reporter assay.

The 5′-UTR sequence for the Nr1d1 gene was amplified by PCR with specific primers: forward, 5′-GTGCCTCATTTCTCCATCACCTACT-3′; reverse, 5′-GGGAGGAGCCACTAGAGCC-3′. The PCR product was subcloned into a pmirGLO vector (Promega) between SacI and SalI sites downstream of the Renilla luciferase gene. To generate the mutant construct, 3 nucleotides in the target site (CCATTGCC) of the miR-182-5p seed region were mutated (CCAATCCT). HEK293T cells (ATCC) were transfected with Nr1d1 5′-UTR luciferase reporter or mutant or blank vector alone with miR-182-5p mimic mixture or mimic controls using Lipofectamine 3000. Cells were collected and firefly and Renilla luciferase activities were measured with a Dual-Glo luciferase assay system (E2940, Promega), according to the manufacturer’s instructions. Luciferase activities were calculated as the ratio of firefly to Renilla luminescence and normalized to the average ratio of the blank control.

### Flow cytometry.

Macrophages isolated from sWAT were washed once in ice-cold flow-staining buffer (PBS, 2% FBS; filter sterilized) and resuspended in flow-staining buffer at a final concentration of 5 × 10^5^ cells. Cells were labeled with the antibodies, incubated in the dark at room temperature for 30 minutes, washed twice, and resuspended in ice-cold staining buffer. Relevant single isotype controls were used. Data were collected and analyzed. M1 and M2 macrophages were identified as F4/80^+^CD11b^+^CD11c^+^ and F4/80^+^CD11b^+^CD206^+^, respectively.

### OCR analysis.

Mitochondrial OCR in intact cells was measured using Seahorse Bioscience XF24 analyzer as described in the manufacturer’s instructions. Briefly, adipose tissues were isolated from mice treated with miR-182-5p agomir or scrambled controls. OCR was determined according to the procedure of Kiefer et al. (35). In brief, freshly isolated sWAT was rinsed with Krebs-Henseleit solution, and the tissues in the absence of large vessels were cut into small pieces and washed extensively. Approximately 10 mg of tissue was placed in each well of an XF24-well Islet Flux plate (Seahorse Bioscience) and covered with a customized screen that allows free perfusion while minimizing tissue movement. Basal OCRs were measured in all wells by an XF24 extracellular flux analyzer, and the data were normalized to tissue weight.

### Data and code availability.

The data that support the findings of this study are available from the corresponding author upon reasonable request. RNA-Seq data generated in this paper can be accessed at the National Center for Biotechnology Information’s Gene Expression Omnibus repository (accession number: GSE178952).

### Statistics.

Statistical analysis was performed using SPSS software version 19.0 (SPSS Inc.) or Microsoft Excel. Statistical parameters, including the statistical test used, exact value of *n*, what *n* represents, and measures of distribution and deviation, are reported in the figure legends. Data analysis involved unpaired 2-tailed Student’s *t* test for 2 groups and 1-way ANOVA for more than 2 groups. Data shown are average ± SEM. *P* ≤ 0.05 was considered statistically significant.

### Study approval.

All animal studies were performed under a protocol approved by the Institutional Animal Care and Use Committee of Central South University (approval CSU: 2020406) and by the Institutional Animal Care and Use Committee at UTHSA (approval UHS: 20200038AR). All animal studies were performed in accordance with the *Guidelines for the Care and Use of Laboratory Animals* of the NIH (National Academies Press, 2011). The study protocol for humans was approved by the IRB of the University of Texas Health Science Center (approval UHS: 20160323N), and all patients provided written informed consent for inclusion. Anesthetization of mice was conducted with isoflurane, and euthanasia was performed with CO_2_ followed by cervical dislocation.

## Author contributions

WM and JB collected and assembled data and prepared the first draft of the manuscript; TX, XL, J Wen, XP, J Wang, Y Zou, JL, CB, HL, Y Zhang, BL, and JZ collected data; FH, ML, LQD, and ZZ supervised students and contributed to data analysis and discussion; and FL and JB contributed to conceptualization and design, data analysis and interpretation, manuscript writing, financial support, and final approval of manuscript. All authors reviewed and approved the manuscript. FL and JB are the guarantors of this work and, as such, had full access to all the data in the study and take responsibility for the integrity of the data and the accuracy of the data.

## Supplementary Material

Supplemental data

## Version 1 07/15/2021

In-Press Preview

## Version 2 09/08/2021

Electronic publication
