# Supplementary material for: The miR-182-5p/FGF21/acetylcholine axis mediates the crosstalk between adipocytes and macrophages to promote beige fat thermogenesis
Source: JCI Insight. 2021 Sep 8;6(17):e150249. doi: 10.1172/jci.insight.150249 (PMC8492300; doi:10.1172/jci.insight.150249)
Supplement: Supplemental data [file jciinsight-6-150249-s217.pdf]

Supplementary Information

The miR-182-5p/FGF21/acetylcholine axis mediates the crosstalk between adipocytes and macrophages to promote beige fat thermogenesis

Wen Meng [1,#], Ting Xiao[1,#], Xiuci Liang[1], Jie Wen[1,2] , Xinyi Peng[1,2], Jing Wang[1], Yi Zou[3], Jiahao Liu[1], Christie Bialowas[4], Hairong Luo[1], Yacheng Zhang[1,5], Bilian Liu[1], Jingjing Zhang[1], Fang Hu[1], Meilian Liu[1,6], Lily Q. Dong[7], Zhiguang Zhou[1], Feng Liu[1,2,*] and Juli Bai[1,2,*]

[1]National Clinical Research Center for Metabolic Diseases, Metabolic Syndrome Research Center, and Department of Metabolism and Endocrinology, The Second Xiangya Hospital of Central South University, Changsha 410011, Hunan, China.
Departments of [2]Pharmacology, [3]Greehey Children's Cancer Research Institute, and [7]Cell Systems & Anatomy, University of Texas Health at San Antonio, San Antonio, Texas, USA
[4]Division of Plastic Surgery, Department of Surgery, Albany Medical Center, Albany, NY 12208-3403
[5]Department of Biochemistry and Molecular Biology, School of Basic Medical Sciences, Xinjiang Medical University, Urumqi, Xinjiang, China
[6]Department of Biochemistry and Molecular Biology, University of New Mexico Health Sciences Center, Albuquerque, New Mexico, USA

*Corresponding author: E-mail: liuf@hotmail.com or baij@uthscsa.edu
Mailing address: Department of Pharmacology, University of Texas Health San Antonio, 7703 Floyd Curl Drive, San Antonio, Texas, 78229.
Juli Bai Phone: 210-567-4526; Email: baij@uthscsa.edu;
Feng Liu: 210-567-3097; Email: liuf@hotmail.com
[#]These authors contribute equally to the work

Running title: miR-182-mediated immune regulation of beige fat development

Word Count: 7314 words.
Number of Figures: 6
Number of Tables: 0

Supplementary Figure 1

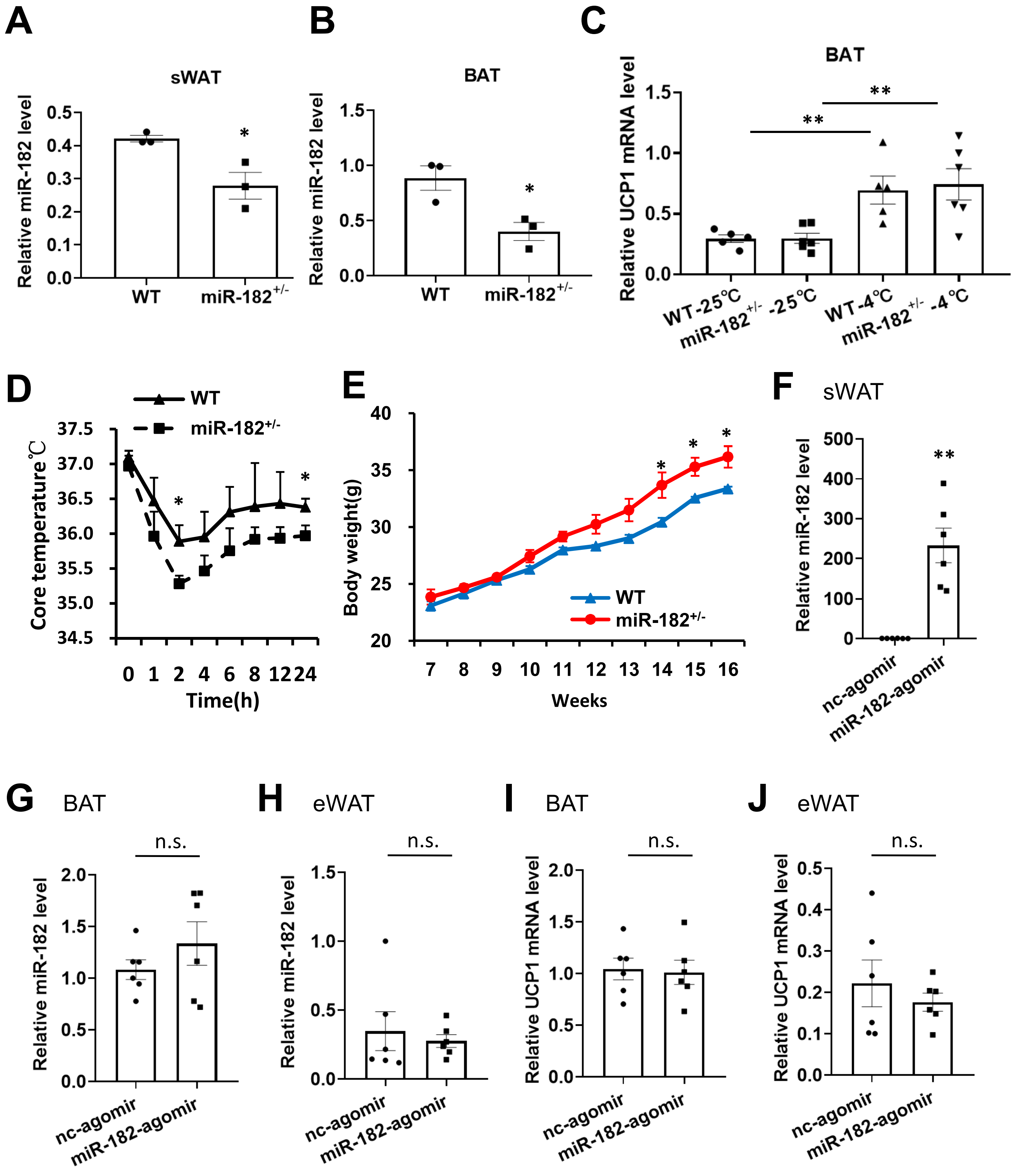

Figure S1. miR-182-5p administration into mouse sWAT had no effect on thermogenic gene expression in BAT and eWAT, Related to Figure 2. The mRNA levels of miR-182-5p in sWAT (A) and BAT (B) of male heterozygous miR-182-5p$^{+/-}$ mice and their wild-type (WT) littermates (n=3/group). Data were mean ± SEM (*P < 0.05). Male miR-182-5p heterozygous knockout mice (miR-182-5p$^{+/-}$) and their wild-type (WT) littermates (8-weeks old) were housed individually in cages and exposed to cold (4 ℃; 6 hours/day) for 7 days. (C) The level of UCP1 mRNA were determined by qRT-PCR in BAT of mice (n=5-6/group). (D) Body temperature of 12-week-old miR-182-5p$^{+/-}$ and WT control mice exposed to cold (4°C) on the ND feeding condition (n=4/group). (E) Body weight of miR-182-5p$^{+/-}$ and WT control mice fed with HFD for 16-week (n=4/group). (F) The level of miR-182-5p was determined by qRT-PCR in sWAT of mice with fat-pad injection of miR-182-5p agomir- or control agomir (n=6/group). The levels of miR-182-5p (G) and UCP1 mRNA (I) were determined by qRT-PCR in BAT of mice with fat-pad injection of miR-182-5p agomir- or control agomir (n=6/group). miR-182-5p levels (H) and the mRNA levels of UCP1 (J) were determined by qRT-PCR in eWAT of mice with fat-pad injection of miR-182-5p agomir- or control agomir (n=6/group). Data represent mean ± SEM. Significance were determined by unpaired 2-tailed Student's *t* test (A-B and D-F) and by one-way ANOVA (C). *P< 0.05, **P<0.01, n.s.: not significant.

Supplementary Figure 2

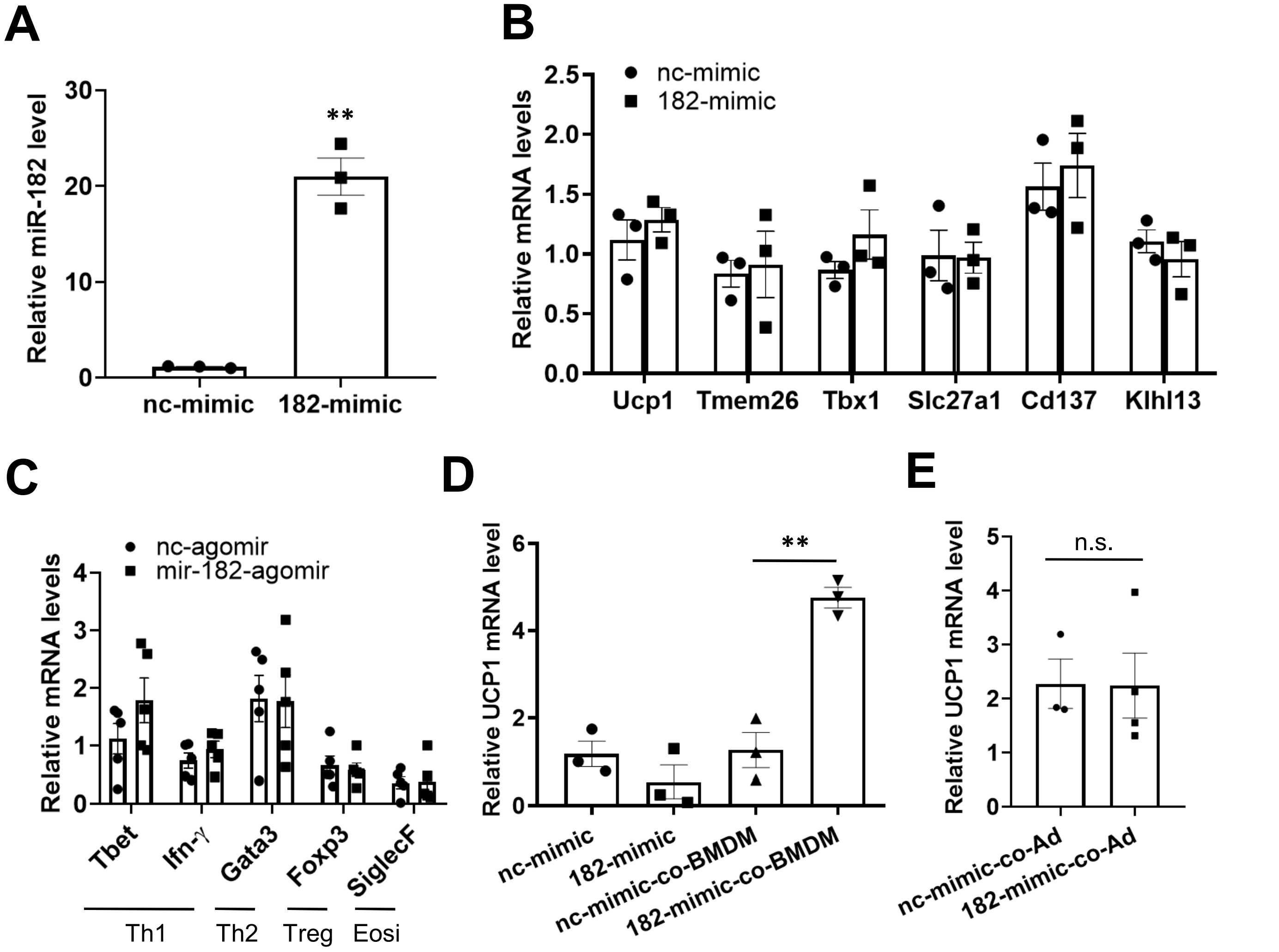

Figure S2. miR-182-5p promotes thermogenic gene expression in adipocytes via a macrophage-dependent mechanism, Related to Figure 3. miR-182-5p mimic (182-mimic) or its negative control (nc-mimic) were overexpressed in mouse primary adipocytes. The cells were harvested and miRNA-182-5p levels (A) and the mRNA levels of thermogenic and beige marker genes (B) were determined by qRT-PCR (n=3/group). (C) The mRNA levels of immune cells marker genes were quantified by qRT-PCR in sWAT of miR-182-5p agomir- or control agomir-injected fat pad mice(n=5/group). miR-182-5p mimic (182-mimic) or its negative control (nc-mimic) were overexpressed in mouse primary adipocytes. The cells were cultured alone or co-cultured with bone marrow-derived macrophages (BMDM) for 3 days (n=3/group). (D) UCP1 mRNA levels were determined by qRT-PCR in primary adipocytes cultured alone or co-cultured with bone marrow-derived macrophages, respectively. (E) miR-182-5p mimic (182-mimic) or its negative control (nc-mimic) were overexpressed in mouse peritoneal macrophages (PM) .The cells were cultured alone or co-cultured with mouse primary adipocytes (Ad) for 3 days (n=3-4/group). The mRNA levels of UCP1 were determined by qRT-PCR in primary adipocyte. Data represent mean ± SEM. Significance were determined by unpaired 2-tailed Student's *t* test (A) and by one-way ANOVA (D). ***P*<0.01, n.s.: not significant.

Supplementary Figure 3

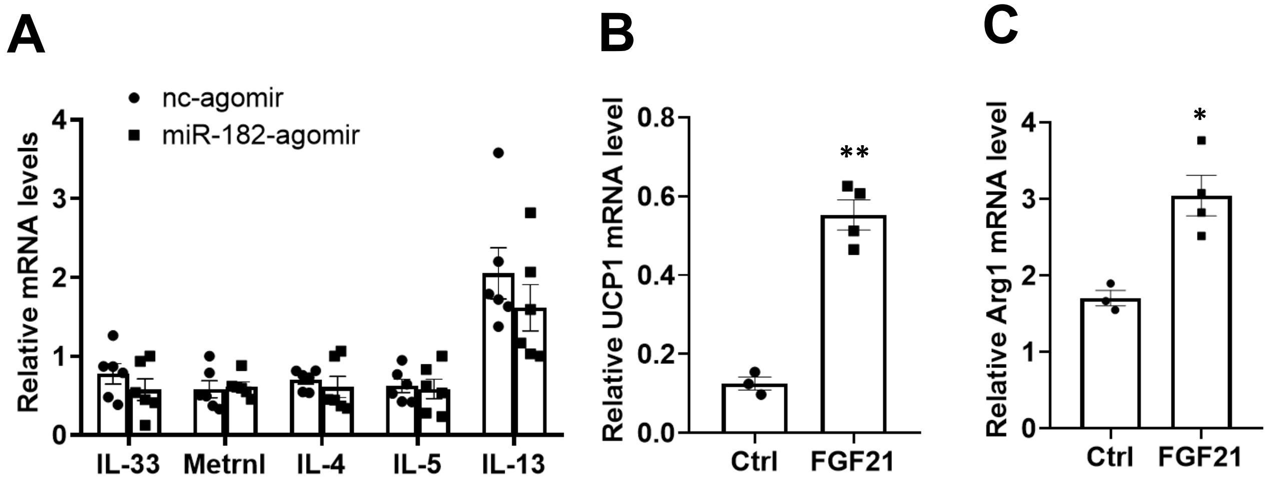

Figure S3. FGF21 promotes UCP1 expression in adipocytes via a macrophage-dependent mechanism, Related to Figure 4. (A) The mRNA levels of interleukins genes were quantified by qRT-PCR in sWAT of miR-182-5p agomir- or control agomir-injected fat pad mice (n=6/group). Bone marrow-derived macrophages (BMDM) were treated with or without FGF21 for 48 hours and condition media (CM) of the cells were collected. Primary adipocytes were incubated with FGF21-treated or -non-treated CM from BMDM cells for 3 days (n=3-4/group). The mRNA levels of UCP1 (B) or the M2 marker gene Arg1(C) were determined by qRT-PCR analysis. Data represent mean ± SEM. Comparisons were made using unpaired 2-tailed Student's *t* test. *$P< 0.05$, **$P<0.01$, n.s.: not significant.

Supplementary Figure 4

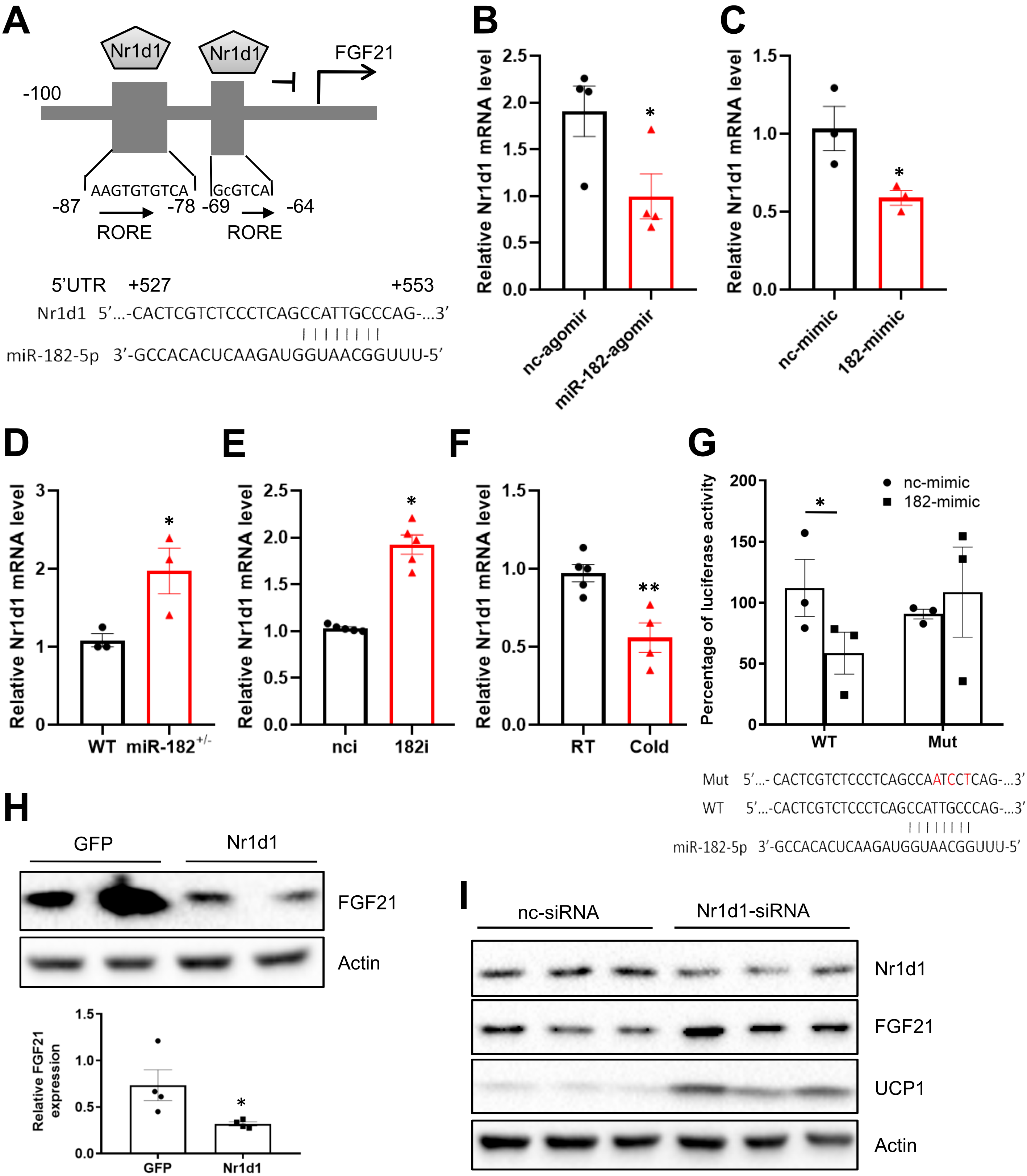

J

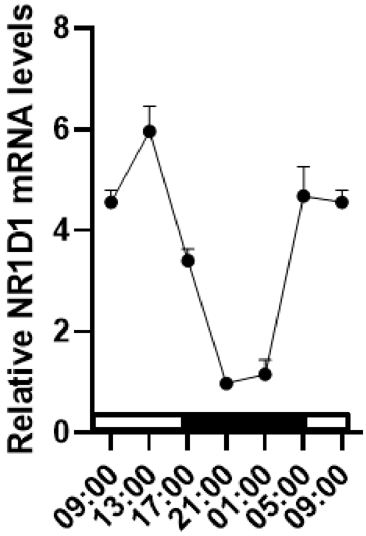

K

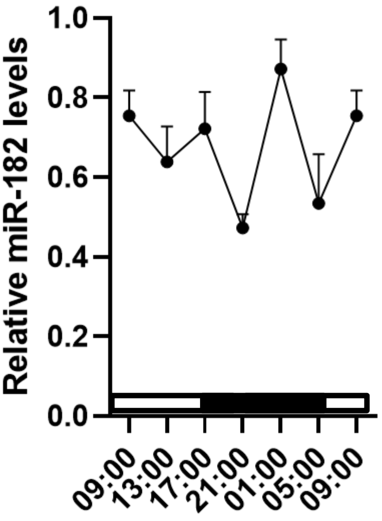

Figure S4. miR-182-5p Increases FGF21 Levels by Suppressing Nr1d1 Gene Expression in sWAT, Related to Figure 4. (A) Schematic diagram of Nr1d1 binding to the consensus RORE half sites of mouse FGF21 promoter sequences. The RNA22 software was used to predict miR-182-5p binding site on Nr1d1 gene. Nr1d1 sequence (mm10, chr11:98767932-98775377) was retrieved from UCSC Genome Browser, while miR-182-5p sequence was retrieved from miRBase. The Nr1d1 mRNA levels in sWAT of miR-182-5p agomir-treated mice (B) and in miR-182-5p overexpressing primary white adipocytes (C) were determined by qRT-PCR analysis (n=3-4/group). qRT-PCR analysis for the Nr1d1 mRNA levels in sWAT of miR-182-5p$^{+/-}$ mice (D) and primary white adipocytes treated with or without miR-182-5p inhibitor (182i) (E) (n=3-5/group). (F) The mRNA level of Nr1d1 in sWAT of C57BL/6 mice maintained at Room temperature (RT; 25° C) or exposed to 4° C (Cold) for one week (n=5/group). (G) Luciferase reporter assay to examine the interactions between miR-182-5p and the predicted target site in the Nr1d1 5‘UTR. Plasmids with the Nr1d1 5’UTR s or mutated UTRs were co-transfected with miR-182-5p mimic or a control mimic into primary white adipocytes. Renilla luciferase activity was measured by a Dual-Glo luciferase assay system and normalized to internal control firefly luciferase activity (n=3 biological Replicates). (H) The FGF21 protein levels in primary white adipocytes transduced with an adenovirus overexpressing Nr1d1 or GFP (control) (n=4 biological Replicates). Values are shown as mean ± SEM. Comparisons were made using paired 2-tailed Student’s t test (B-H) (I) Nr1d1, FGF21 and UCP1 protein levels in Nr1d1-siRNA- or negative control siRNA (nc-siRNA)- treated primary white adipocytes co-cultured with PMs were determined by Western blot (n=3/group). NR1D1 gene(J) and miR-182-5p (K) expression in sWAT of mice over a 24h period. Data represent mean ± SEM. Comparisons were made using unpaired 2-tailed Student’s *t* test. *P< 0.05, **P<0.01.

Supplementary Figure 5

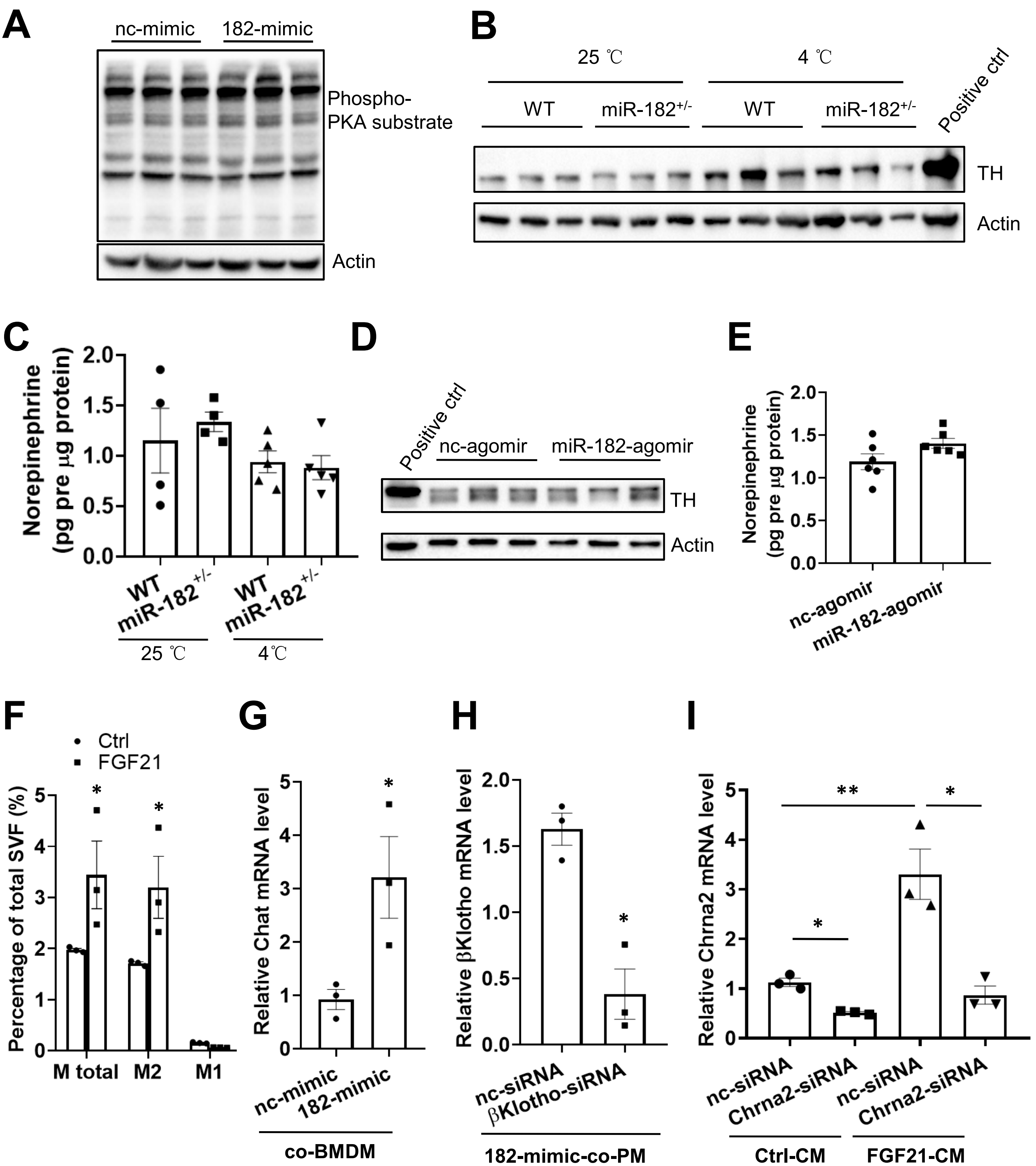

Figure S5. The stimulatory effect of miR-182-5p on UCP1 expression is mediated by a NE release-independent mechanism, Related to Figure 5. (A) PKA substrate phosphorylation in primary white adipocytes overexpressing miR-182-5p mimic (182-mimic) or its negative control (nc-mimic) (n=3/group). (B) Tyrosine hydroxylase (Th) expression in sWAT of miR-182-5p$^{+/-}$ and WT control mice was determined by WB (n=3/group). (C) NE levels in sWAT of miR-182-5p$^{+/-}$ and WT control mice were determined by ELISA (n=4-5/group). (D) Tyrosine hydroxylase (Th) expression in sWAT of mice injected in fat pad with miR-182-5p agomir- or control agomir was determined by WB (n=3/group). (E) NE levels in sWAT of mice injected with miR-182-5p agomir or control agomir in the fat pad were determined by ELISA (n=6/group). (F) Flow cytometry analysis showing the percentage of total, M1, and M2 macrophages in SVF-derived white preadipocytes treated with or without FGF21 (n=3/group). (G) The mRNA level of Chat was quantified by qRT-PCR in BMDM co-cultured with primary adipocytes overexpressing miR-182-5p (n=3/group). (H) βKlotho mRNA levels in βKlotho-siRNA- or negative control siRNA (nc-siRNA)- treated PMs co-cultured with primary white adipocytes overexpressing miR-182-5p mimic were determined by qRT-PCR (n=3/group). (I) PM cells were treated with or without FGF21 for 48 hours and condition media (CM) were collected. Primary adipocytes transfected Chrna2 siRNA or its negative control siRNA were incubated with the FGF21-treated or non-treated conditioned medium (CM) for 3 days (n=3/group). Chrna2 mRNA levels in primary adipocytes incubated with CM from PM treated with or without FGF21 was determined by qRT-PCR. Data represent mean ± SEM. Significance were determined by unpaired 2-tailed Student's *t* test (F-H) and by one-way ANOVA (I). **P*< 0.05, ***P*<0.01, n.s.: not significant.

Supplementary Figure 6

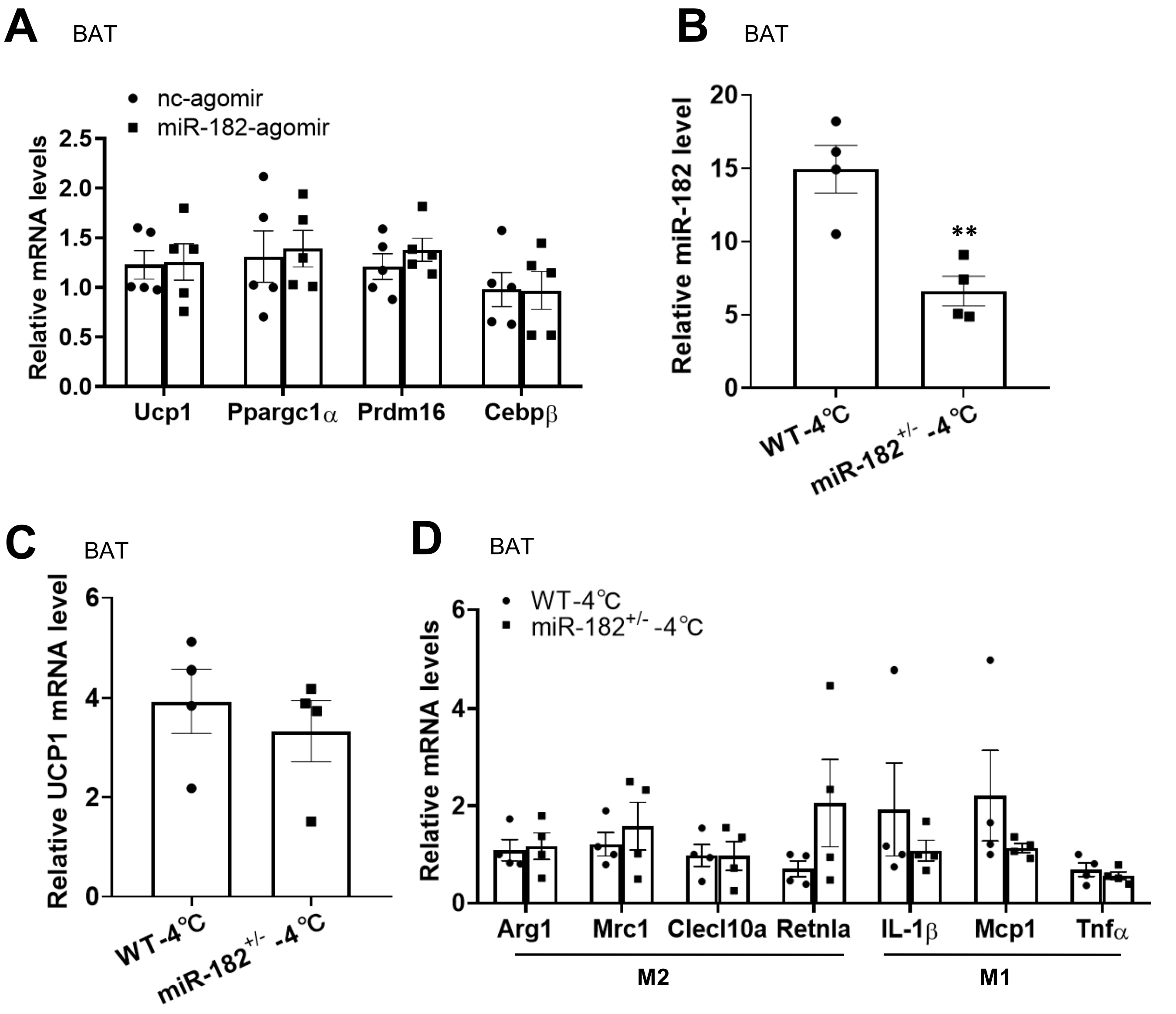

Figure S6. miR-182-5p deficiency had no effect on Ucp1 expression and macrophages polarization in BAT of cold-exposed mice, Related to Discussion. (A) mRNA levels of thermogenic marker genes in BAT of HFD-fed miR-182-5p-agomir and nc-agomir mice were quantified by qRT-PCR and normalized to β-actin (n=5/group). Male miR-182-5p$^{+/-}$ mice and their wild-type (WT) littermates (8-weeks old) housed individually in cages were exposed to cold (4 ℃; 6 hrs/day) for 7 days (n=4/group). miR-182-5p levels (B) and UCP1 mRNA levels (C) or M1 and M2 macrophage marker genes (D) in BAT were determined by qRT-PCR. Data represent mean ± SEM. Comparisons were made using unpaired 2-tailed Student's *t* test. ***P*<0.01, n.s.: not significant.

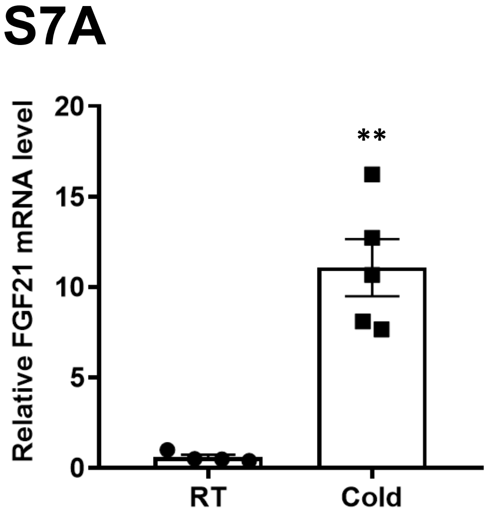

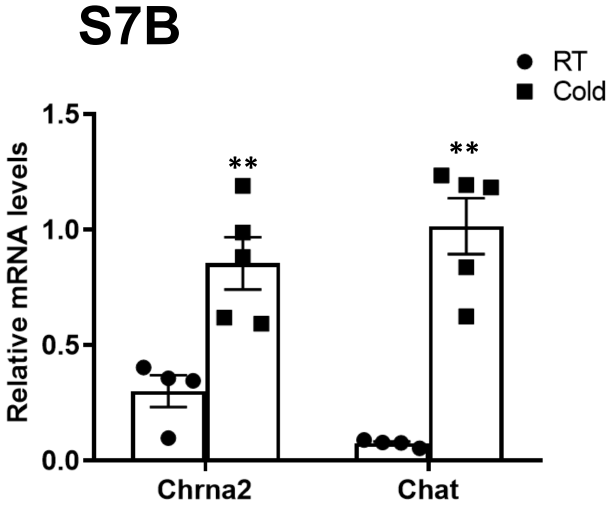

Figure S7. The expression levels of FGF21/Chat/Chrna2 are induced in sWAT of cold-exposed mice, Related to Discussion. The mRNA levels of FGF21(A) and Chat or Chrna2 (B) in sWAT of mice exposed to cold (4℃; 6 hrs/day) or maintained at room temperature (RT) for 7 days were determined by qRT-PCR (n =4- 5/group). Data represent mean ± SEM. Comparisons were made using unpaired 2-tailed Student's *t* test. $^{**}P<0.01$.
